# The interplay of homology‐directed repair pathways in the repair of zebularine‐induced DNA–protein crosslinks in Arabidopsis

**DOI:** 10.1111/tpj.16863

**Published:** 2024-06-02

**Authors:** Eva Dvořák Tomaštíková, Jitka Vaculíková, Veronika Štenclová, Kateřina Kaduchová, Zuzana Pobořilová, Jan J. Paleček, Ales Pecinka

**Affiliations:** ^1^ Centre of Plant Structural and Functional Genomics, Institute of Experimental Botany of the Czech Academy of Sciences Šlechtitelů 31 Olomouc 77900 Czech Republic; ^2^ Faculty of Science National Center for Biomolecular Research, Masaryk University Kamenice 5 Brno 62500 Czech Republic; ^3^ Mendel Centre for Plant Genomics and Proteomics Central European Institute of Technology, Masaryk University Kamenice 5 Brno 62500 Czech Republic; ^4^ Present address: Department of Immunology, Faculty of Medicine and Dentistry Palacky University and University Hospital Olomouc Czech Republic

**Keywords:** DNA damage repair, replicative stress, DNA–protein crosslink, RTEL1, TEBICHI, SMC5/6 complex, genome stability, *Arabidopsis thaliana*

## Abstract

DNA–protein crosslinks (DPCs) are highly toxic DNA lesions represented by proteins covalently bound to the DNA. Persisting DPCs interfere with fundamental genetic processes such as DNA replication and transcription. Cytidine analog zebularine (ZEB) has been shown to crosslink DNA METHYLTRANSFERASE1 (MET1). Recently, we uncovered a critical role of the SMC5/6‐mediated SUMOylation in the repair of DPCs. In an ongoing genetic screen, we identified two additional candidates, *HYPERSENSITIVE TO ZEBULARINE 2* and *3*, that were mapped to *REGULATOR OF TELOMERE ELONGATION 1* (*RTEL1*) and polymerase *TEBICHI* (*TEB*), respectively. By monitoring the growth of *hze2* and *hze3* plants in response to zebularine, we show the importance of homologous recombination (HR) factor RTEL1 and microhomology‐mediated end‐joining (MMEJ) polymerase TEB in the repair of MET1‐DPCs. Moreover, genetic interaction and sensitivity assays showed the interdependency of SMC5/6 complex, HR, and MMEJ in the homology‐directed repair of MET1‐DPCs in Arabidopsis. Altogether, we provide evidence that MET1‐DPC repair in plants is more complex than originally expected.

## Significance Statement

DNA–protein crosslinks (DPCs) are highly toxic DNA lesions represented by proteins covalently bound to the DNA. We show the importance of homologous recombination factor *RTEL1* and microhomology‐mediated end joining polymerase *TEB* and their interdependency with the SMC5/6 complex in the repair of DNA METHYLTRANSFERASE1‐DPCs. This suggests a complex network of pathways detoxifying DPCs in plants.

## INTRODUCTION

Cellular DNA is constantly exposed to genotoxic factors of endogenous and exogenous origin, which cause its damage. The lesions need to be repaired to avoid DNA damage‐induced blockage of replication and transcription, chromosome breakage, and changes or loss of genetic information. Organisms have developed DNA damage repair mechanisms specialized for the removal of diverse types of DNA damage.

DNA–protein crosslinks (DPCs) are a specific type of damage where DNA and a nearby protein become covalently bound. It is a diverse group of lesions, varying based on the size and type of protein involved, the chemical nature of the covalent bonds, and the associated type of DNA strand break. Based on the principle of covalent bond formation, two types of DPCs are recognized. Non‐enzymatic DPCs form between DNA and any nearby protein and arise spontaneously in cells due to metabolic by‐products or can be caused by exposure to external factors like UV‐B radiation or ionizing radiation in hypoxic conditions. On the contrary, enzymatic DPCs arise when an enzyme, typically associated with DNA as a part of its metabolic cycle, becomes covalently attached to the DNA molecule (reviewed in Weickert & Stingele, 2022). The best‐described model systems to study DPCs include enzymatic DPCs of TOPOISOMERASES. They are generated by camptothecin (CPT) that crosslinks TOPOISOMERASE 1 (TOP1) (Pommier & Marchand, 2011) and by dexrazoxane (ICRF‐187) that crosslinks TOPOISOMERASE 2 (TOP2) (Classen et al., 2003; Lee et al., 2017).

We described cytidine analog zebularine (ZEB) as a potent enzymatic DNA–protein crosslinker in Arabidopsis (Prochazkova et al., 2022). ZEB is chemically similar to 5‐azacytidine but has greater chemical stability in aqueous solutions. It is metabolized in a complex manner and part of the variants is incorporated into DNA during replication. We recently showed that ZEB covalently traps DNA METHYLTRANSFERASE 1 (MET1) and causes massive accumulation of its DPCs at repetitive *45S rDNA* arrays (Prochazkova et al., 2022). There are hundreds of copies of *45S rDNA* in Arabidopsis divided into two similarly sized loci on the top arms of chromosomes 2 and 4 (Copenhaver & Pikaard, 1996a, 1996b; Sims et al., 2021). As only a fraction of the copies on chromosome 4 is transcriptionally active in somatic tissues (Fultz et al., 2023; Rabanal et al., 2017), most of the *45S rDNA* are organized into compact heterochromatic chromocenters (Fransz et al., 2002). Therefore, zebularine MET1 DPCs represent a unique type of DNA damage in at highly repetitive heterochromatic environment.

Homologous recombination (HR) is a repair mechanism that deals with various lesions, including DNA double‐strand breaks (DSBs) or blocked replication forks. During the initial stages of HR, DSBs are processed, generating 3′‐end single‐stranded DNA (ssDNA) tails that invade homologous sequence on an intact chromosome, forming a displacement loop (D‐loop). Then, DNA polymerases synthesize new DNA strand(s), and different regulators direct different outcomes of these processes (reviewed in Krejci et al., 2012). For example, the disruption of D‐loops, following extension by DNA polymerase, leads to the synthesis‐dependent strand annealing (SDSA), the preferred mode of HR in somatic cells, leading to an obligatory non‐crossover outcome (reviewed in Wright et al., 2018). An alternative DSB repair pathway is single‐strand annealing (SSA), involving dispersed or tandem repetitive sequences that flank a single DSB. REGULATOR OF TELOMERE ELONGATION 1 (RTEL1) is a highly conserved SUPERFAMILY2 (SF2) class helicase, which is able to disrupt nascent D‐loops through the unwinding of double‐stranded DNA (dsDNA) in an ATP‐dependent manner (Barber et al., 2008) and stimulate SDSA pathway. Notably, RTEL1 also plays a role in resolving other complex DNA secondary structures arising from DNA replication (Hu et al., 2015; Vannier et al., 2013). Its loss in Arabidopsis leads to uncontrolled HR, accumulation of replication stress (Hu et al., 2015), instability of *45S rDNA* repeats (Dorn et al., 2019; Röhrig et al., 2016), and compromised telomere stability (Olivier et al., 2018).

Replicative DNA polymerases are highly accurate enzymes, but they can become stalled at sites of DNA damage (Johansson & Dixon, 2013). POLYMERASE THETA (POL θ, also known as TEBICHI or TEB) plays a prominent role in repairing DNA damage associated with replication (Inagaki et al., 2006; Klemm et al., 2017; Nisa et al., 2021). TEB comprises helicase and DNA polymerase domains, and in addition to its functions in genome stability, it plays a role in cell division and differentiation (Inagaki et al., 2006, 2009). TEB is involved in microhomology‐mediated end‐joining repair pathway (MMEJ; also known as alternative non‐homologous end‐joining [NHEJ] repair pathway) and, together with the classical NHEJ pathway, plays a central role in DSB repair in plants (Merker et al., 2024). MMEJ uses microhomology regions that serve as templates for DNA synthesis to fill the break (Gehrke et al., 2022). Additionally, TEB has been shown to have a translesion synthesis (TLS) activity that helps to overcome various DNA damages (Gehrke et al., 2022; Merker et al., 2024; Sakamoto, 2019).

The understudied mechanism of ZEB‐induced DPC repair and the complexity of DNA damage repair pathways prompted us to design a *HYPERSENSITIVE TO ZEBULARINE* (*HZE*) forward‐directed genetic screen. The first complementation group, *HZE1*, was mapped to the *STRUCTURAL MAINTENANCE OF CHROMOSOME 6B* (*SMC6B*) subunit of the SMC5/6 complex. We showed that the SMC5/6 works in parallel to known DPC repair pathways, and its SUMOylation activity plays a critical role (Dvořák Tomaštíková, Prochazkova, et al., 2023). Here, we describe two new mapped genes *HZE2* as *RTEL1* and *HZE3* as *TEB*, in which *RTEL1* and *TEB* are mutated. We provide evidence that MET1‐DPCs are not processed only via HR but also by microhomology‐based repair. Importantly, we show that the SMC5/6 complex genetically interacts with RTEL1 and TEB during the repair of ZEB‐induced DPCs.

## RESULTS

### *HZE2/RTEL1* contributes to the repair of zebularine‐induced DPCs

**Figure 1:**
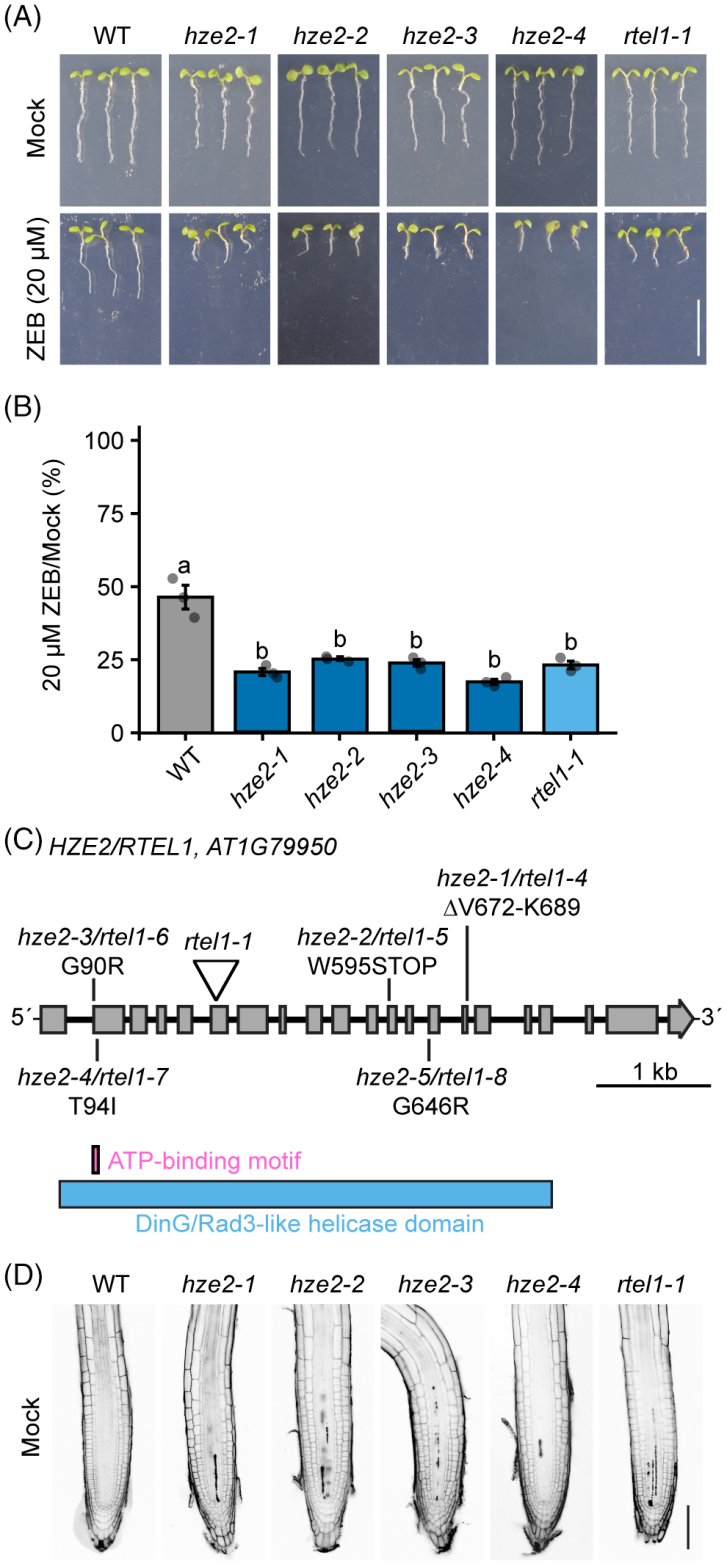
*HYPERSENSITIVE TO ZEBULARINE 2* (*HZE2*) encodes the *REGULATOR OF TELOMERE ELONGATION 1* (*RTEL1*). (A) Representative growth phenotypes of seedlings from wild‐type (WT), *rtel1‐1*, and *hze2* alleles on 0 (mock) and 20 μm zebularine (ZEB). Scale bar, 1 cm. (B) Relative root length of seedlings in (a) under zebularine/mock conditions (*y*‐axis). Data are means ± SD from three biological replicates, each with a minimum of 20 seedlings. Different lowercase letters indicate significant differences (*P* < 0.05), according to one‐way anova followed by Tukey's test. Source data for statistical analyses are available in Table S1. (C) Schematic model of the *RTEL1/HZE2* locus (AT1G79950) with the positions of individual mutations. DinG/Rad3‐like helicase domain (blue) and its highly conserved ATP‐binding motif I (pink) are labeled. (D) Representative confocal microscopy images of root tips stained with propidium iodide, which indicates dead cells (dark sectors). Scale bar, 100 μm.

The *HYPERSENSITIVE TO ZEBULARINE 2* (*HZE2*) mutant candidate *hze2‐1* exhibited approximately 79.5 ± 2.1% reduction in root length on 20 μm zebularine‐containing media compared to 53.6 ± 7.0% decrease in wild type (WT) (Figure 1A,B; Table S1). To identify the causal gene, we performed mapping by sequencing (MBS) using a pool of ~100 zebularine‐sensitive plants from the F2 back‐cross of *hze2‐1* with the parental line. The *hze2‐1* causal mutation localized to the telomere proximal region at the bottom arm of chromosome 1 (Figure S1a,b). Among the moderate‐ to high‐effect mutations, this region contained a G → A transition in the known DNA damage response factor *RTEL1* (AT1G79950) at position Chr1:30077479 (Figure 1C; Figure S1c). This mutation affected the splice donor/acceptor site in the 15th exon, leading to alternative splicing of exon 15 and generating a deletion of 18 amino acids in the C‐terminal end of the DinG/Rad3‐like helicase domain (∆V672‐K689). T‐DNA insertion mutant allele of *RTEL1* (*rtel1‐1*) displayed a similar 76.7 ± 2.3% reduction in root length on 20 μm zebularine‐containing media (Figure 1A,B). We confirmed that *HZE2* is allelic to *RTEL1* by a genetic complementation test showing that F1 *hze2‐1* × *rtel1‐1* hybrids are hypersensitive to zebularine treatments (Figure S2a).

In addition to *hze2‐1*, further screening revealed four more *hze2* alleles, *hze2‐2* to *hze2‐5* (note: *hze2‐1*–*hze2‐5* were marked as *rtel1‐4*–*rtel1‐8*; Figure 1; Figures S3–S6), which were also confirmed through complementation crosses with *rtel1‐1* (Figure S2a). The *hze2‐2* had a G → A transition at position Chr1_30 076 760, resulting in a premature stop codon at W595 (W595*) of the helicase domain (Figure 1C; Figure S3). Interestingly, we identified three missense mutations in the ATP‐binding pocket of the DinG/Rad3‐like helicase domain. The *hze2‐3*, *hze2‐4*, and *hze2‐5* had G → A transitions at positions Chr1_30 074 096; Chr1_30 074 109; and Chr1_30 077 129, resulting in missense mutations of G90R, T94I, and G646R, respectively (Figure 1A–C; Figures S4–S6).

We analyzed root cell viability and the anatomy of the meristematic zone using a propidium iodide (PI) stain (Figure 1D). Under normal conditions, *rtel1‐1* plants accumulate replication‐associated DNA damage, leading to an increased number of dead cells and a shortened root meristematic zone (Hu et al., 2015; Recker et al., 2014). Correspondingly, the *hze2* mutants also exhibited an accumulation of dead cells in the root meristematic zone. It is worth noting that the sensitivity assays with CPT and ICRF‐187 did not reveal a significant increase in these mutants (Figure S7a–d; Table S2). In conclusion, our findings suggest that RTEL1 plays a role in the repair of zebularine‐induced DPCs, but it is not involved in the repair of DPCs caused by CPT and ICRF‐187, respectively.

### RTEL1 plays different roles in homology‐directed repair

**Figure 2:**
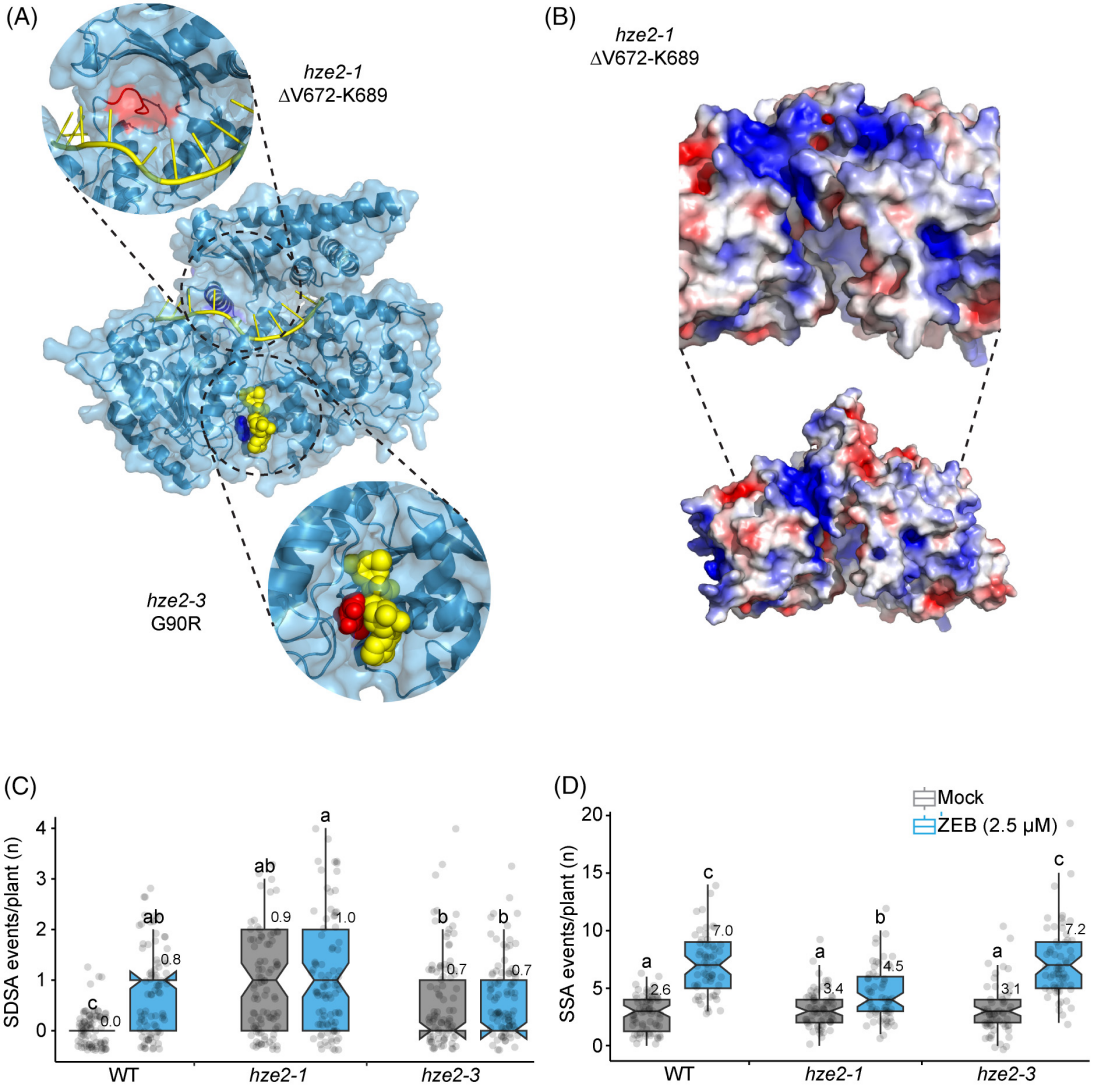
*hze2‐1* and *hze2‐3* mutations change the frequency of somatic homology‐directed repair. (A, B) Structural analyses of *hze2‐1* and *hze2‐3*. (A) Combined surface/cartoon representation (light/dark blue) of the Arabidopsis *At*RTEL1 AlphaFold structure model (aa45‐785). ATP (yellow spheres) and ssDNA (yellow ribbon) molecules were manually docked to the ATP‐binding pocket and DNA‐binding groove, respectively (based on 6FWS crystal structure; Cheng & Wigley, 2018). Small Gly90 residue (dark‐blue spheres; middle panel) was substituted by bulky Arg (red spheres in detail circular window below) as in *hze2‐3* mutant, obstructing ATP binding to the pocket. Deletion of the aa672‐689 residues (dark‐blue helix) in the *hze2‐1* mutant results in ssDNA‐binding groove reshaping (red part in detail circular window above), which could affect RTEL1 binding to the ssDNA. (B) Electrostatic charge distribution at the *At*RTEL1 surface (WT, bottom). The top view [90° rotated compared to panel (A)] suggests a shallow ssDNA‐binding groove present in *hze2‐1* (detail above) compared to a deep groove in WT. (C, D) Wild‐type (WT) and *hze2* plants carrying genomic substrates *IC9C* and *B11* for (C) synthesis‐dependent strand annealing (SDSA) and (D) single‐strand annealing (SSA) types of homology‐directed repair were grown on 0 (Mock) and 2.5 μm zebularine (ZEB)‐containing media. Gray dots indicate the number of repair events per plant. The boxplots' hinges are in the first and third quartile, with a marked median. Whisker marks show the lowest or highest value within the 1.5 interquartile range below or above hinges. Different lowercase letters indicate significant differences between the Kruskal–Wallis *H*‐test with post hoc Conover–Iman test of multiple comparisons with the Benjamini–Hochberg procedure (*P* < 1/2 *a*, *a* = 0.05). Source data for statistical analyses are available in Table S3.

The protein structure modeling mapped the *hze2‐3* (G90R) within the ATP‐binding pocket of the DinG/Rad3‐like helicase domain (Figure 2A). Interestingly, the G90 and T94 (*hze2‐4*) amino acids are highly conserved in the helicase family as they constitute the core of the ATP‐binding pocket (ATP‐binding motif I; Cheng & Wigley, 2018), and their substitutions with bulky residues (like G90R in *hze2‐3*) lead to helicase function impairment (Fan et al., 2008). In comparison, the *hze2‐1* mutation (∆V672‐K689) altered the region responsible for binding to ssDNA (Figure 2B) and may have affected its affinity to ssDNA (Cheng & Wigley, 2018). Therefore, we used *hze2‐1* and *hze2‐3* mutants to analyze different aspects of RTEL1 functions.

RTEL1 has a dual role during HR by blocking spontaneous recombination via RAD51 presynaptic filaments disruption and promoting SDSA via disruption of the D‐loop after extension of DNA (Hu et al., 2015; Recker et al., 2014). To estimate the effect of different RTEL1 functions, we analyzed the frequency of homology‐directed repair (HDR) in *hze2‐1* and *hze2‐3* lines by combining them with reporters *IC9C* and *B11*. Both lines carry the overlapping fragments of the reporter gene *uidA* coding for β‐glucuronidase (GUS). Line *B11* contains the three tandemly organized copies of the reporter gene with the fragments organized in direct orientation GU‐US (1213 overlap), allowing for gene restoration by SSA and in principle also SDSA (Puchta et al., 1995; Swoboda et al., 1994). On the contrary, line *IC9C* carries a single copy of the reporter construct with the US‐GU‐oriented fragments in requiring SDSA for repair (Molinier et al., 2004). In agreement with the published data (Recker et al., 2014), *hze2‐1 IC9C* and *hze2‐3 IC9C* lines exhibited a significantly increased frequency of SDSA (all statistics tested with Kruskal–Wallis *H*‐test with post hoc Conover–Iman test of multiple comparisons with Benjamini–Hochberg procedure, *P* < 0.05) compared to wild‐type *IC9C* plants. On average, there were 0.0 ± 0.0 SDSA events per plant in WT *IC9C* (number of analyzed plants [*n*] = 90), 0.98 ± 0.1 in *hze2‐1 IC9C* (*n* = 91), and 0.7 ± 0.0 in *hze2‐3 IC9C* plants (*n* = 90) under control conditions (Figure 2C; Table S3). This finding highlights the essential role of the RTEL1 helicase domain in the HR repair of DPCs via the SDSA mechanism. Conversely, no significant differences in SSA frequency were observed for *hze2‐1 B11* and *hze2‐3 B11* lines compared to wild‐type *B11* plants under mock conditions. The average number of SSA events per plant was 2.6 ± 0.5 in WT *B11* (*n* = 70), 3.4 ± 0.1 in *hze2‐1 B11* (*n* = 69), and 3.1 ± 0.9 in *hze2‐3 B11* plants (*n* = 60) (Figure 2D; Table S3). Overall, these results confirm the role of RTEL1 in blocking spontaneous HR and show the specific function of RTEL1 in the SDSA pathway.

Using genetic analyses, previous studies have demonstrated that zebularine‐induced DPCs are repaired through both SSA and SDSA (Liu et al., 2015; Nowicka et al., 2020). Therefore, we tested the dependency of the repair of ZEB‐induced DPCs on RTEL1 on 10‐day‐old plants exposed to 2.5 μm ZEB as milder genotoxic stress allows the seedlings to grow on the chemical for a longer period of time (10 days). A significant increase in the repair frequency was observed in WT *IC9C* and *B11* plants after ZEB treatment compared to mock conditions. There were 0.8 ± 0.12 SDSA events per plant in WT *IC9C* (*n* = 85) and 7.0 ± 0.2 SSA events in WT *B11* (*n* = 60) (Figure 2C; Table S3). On the one hand, no significant increase in SDSA frequency was observed in ZEB‐treated *hze2‐1 IC9C* and *hze2‐3 IC9C* mutants compared to mock‐treated plants and WT *IC9C* controls. There were 1.0 ± 0.0 SDSA events per plant in *hze2‐1 IC9C* (*n* = 89), and 0.7 ± 0.2 in *hze2‐3 IC9C* (*n* = 90) (Figure 2C; Table S3). On the other hand, a significant increase in the SSA frequency was found for both *hze2‐1 B11* and *hze2‐3 B11* mutants after ZEB treatment compared to mock conditions. However, there were 4.5 ± 0.7 SSA events per plant in *hze2‐1 B11* (*n* = 60), while 7.0 ± 0.2 in WT *B11* and 7.2 ± 1.4 in *hze2‐3 B11* (*n* = 60) (Figure 2D; Table S3), suggesting reduced efficiency of SSA in ssDNA‐binding defective *hze2‐1* mutant. This indicates that under normal conditions, both ATP‐ and ssDNA‐binding activities of *RTEL1* play a role in suppressing unwanted HR through the SDSA mechanism. In agreement with the role of RTEL1 in D‐loop resolution on the extended DNA strand, plants defective in *RTEL1* ATP‐ and ssDNA‐binding activities cannot repair DNA damage through SDSA. In contrast, both RTEL1 activities were dispensable for SSA under normal conditions, while the ssDNA‐binding defect in *hze2‐1* partially compromised the SSA efficiency of zebularine‐induced damage repair.

### Polymerase TEBICHI participates in the repair of various types of DPCs

**Figure 3:**
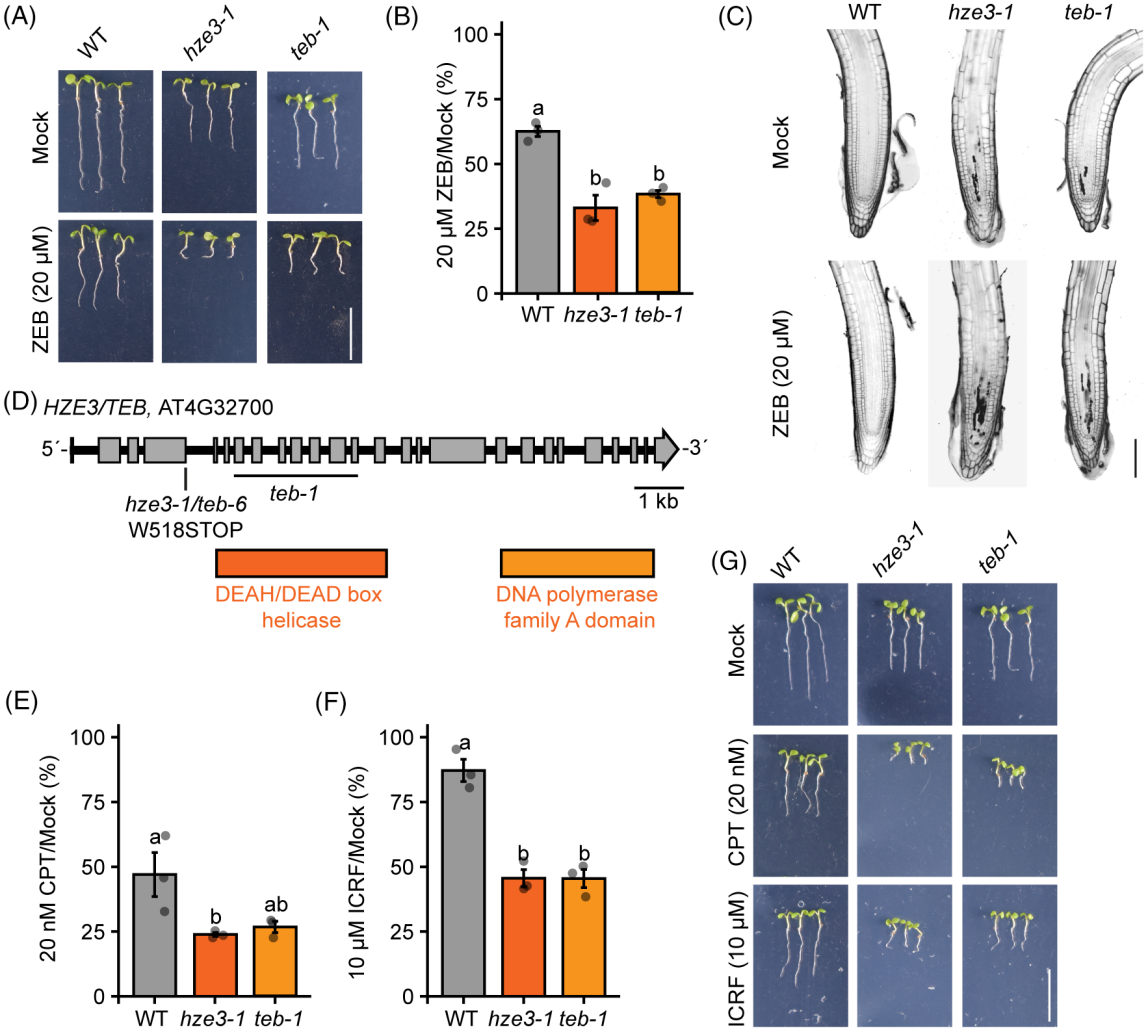
*HYPERSENSITIVE TO ZEBULARINE 3* (*HZE3*) encodes *TEBICHI* (*TEB*). (A) Representative growth phenotypes of seedlings from wild‐type (WT), *teb‐1*, and *hze3‐1* on 0 (Mock) and 20 μm zebularine (ZEB). Scale bar, 1 cm. (B) Relative root length of seedlings in (A) under zebularine/mock conditions (% of ZEB/Mock). Data are means ± SD from three biological replicates, each with a minimum of 20 seedlings. Different lowercase letters indicate significant differences (*P* < 0.05), according to one‐way anova followed by Tukey's test. Source data for statistical analyses are available in Table S4. (C) Representative confocal microscopy images of root tips stained with propidium iodide, which indicates dead cells (dark sectors). Five‐day‐old seedlings were treated with 20 μm ZEB for 24 h prior to analysis. Scale bar, 100 μm. (D) Schematic model of the *TEB/HZE3* locus (AT4G32700) with the positions of individual mutations. (E, F) Relative root length of seedlings in (G) under camptothecin [(E), % of CPT/Mock] and ICRF‐187 [(F), % of ICRF/Mock] conditions. Data are means ± SD from three biological replicates, each with a minimum of 20 seedlings. Statistical analyses as in (B). Source data for statistical analyses are available in Table S4. (G) Representative growth phenotypes of seedlings from wild‐type (WT), *teb‐1*, and *hze3‐1* allele on 0 (Mock), 20 nm camptothecin (CPT), and 10 μm ICRF‐187 (ICRF). Scale bar, 1 cm.

Furthermore, we identified another mutant candidate *hze3‐1*, showing 67.0 ± 8.5% reduction in root length on 20 μm zebularine relative to mock conditions (Figure 3A,B; Table S4). These plants exhibited an increased number of dead cells already under normal conditions (Figure 3C), but lacked serrated leaves typical for *RTEL1* mutants, suggesting it is a new candidate.

We performed MBS and identified candidate regions at the bottom arm of chromosome 1 and at chromosome 4 (Figure S8a). Detailed analyses of both regions for moderate‐ to high‐effect genic mutations revealed G → A transition at position Chr4_15 769 749 that resulted in the premature stop codon at W518 in gene AT4G32700 annotated as *POLYMERASE THETA/TEBICHI* (*POLQ*, *TEB*; Figure 3D; Figure S8b,c). TEBICHI is involved in MMEJ in DSB repair and TLS during replication (Inagaki et al., 2006), making it our primary candidate. Complementation tests between *hze3‐1* and *teb‐1* confirmed that these mutations are allelic (Figure S2b). Therefore, we marked this mutation as *hze3‐1/teb‐6*.

We explored whether *TEBICHI* is involved in the repair of other types of DPCs. First, we analyzed the sensitivity of *TEB* mutants to CPT, which induces Type 2 DPCs. We observed a significantly higher sensitivity (one‐way anova with Tukey post hoc test, *P* < 0.05) in *teb‐1* and *hze3‐1/teb‐6* compared to WT plants (*teb‐1* 26.6 ± 3.8% of mock‐treated plants, *hze3‐1* 23.9 ± 1.3% of mock‐treated plants compared to 47.0 ± 14.7% in WT plants) (Figure 3E,G; Table S4). Next, we analyzed the sensitivity of *TEB* mutants to ICRF‐187 and found a significant reduction in root length in *teb‐1* and *hze3‐1* mutants compared to WT plants (*teb‐1* 45.5 ± 6.1% of mock‐treated plants and *hze3‐1* 45.6 ± 5.7% of mock‐treated plants compared to 87.2 ± 7.4% in WT plants; Figure 3F,G; Table S4). Therefore, unlike RTEL1, TEB contributes to the repair of Type 1, 2, and 4 DPCs. Coupled with its function in the repair of DSBs and DNA intra‐strand crosslinks (Inagaki et al., 2006), TEBICHI is a global player involved in plant DNA damage repair.

### Simultaneous loss of RTEL1 and TEB leads to embryonic lethality

**Figure 4:**
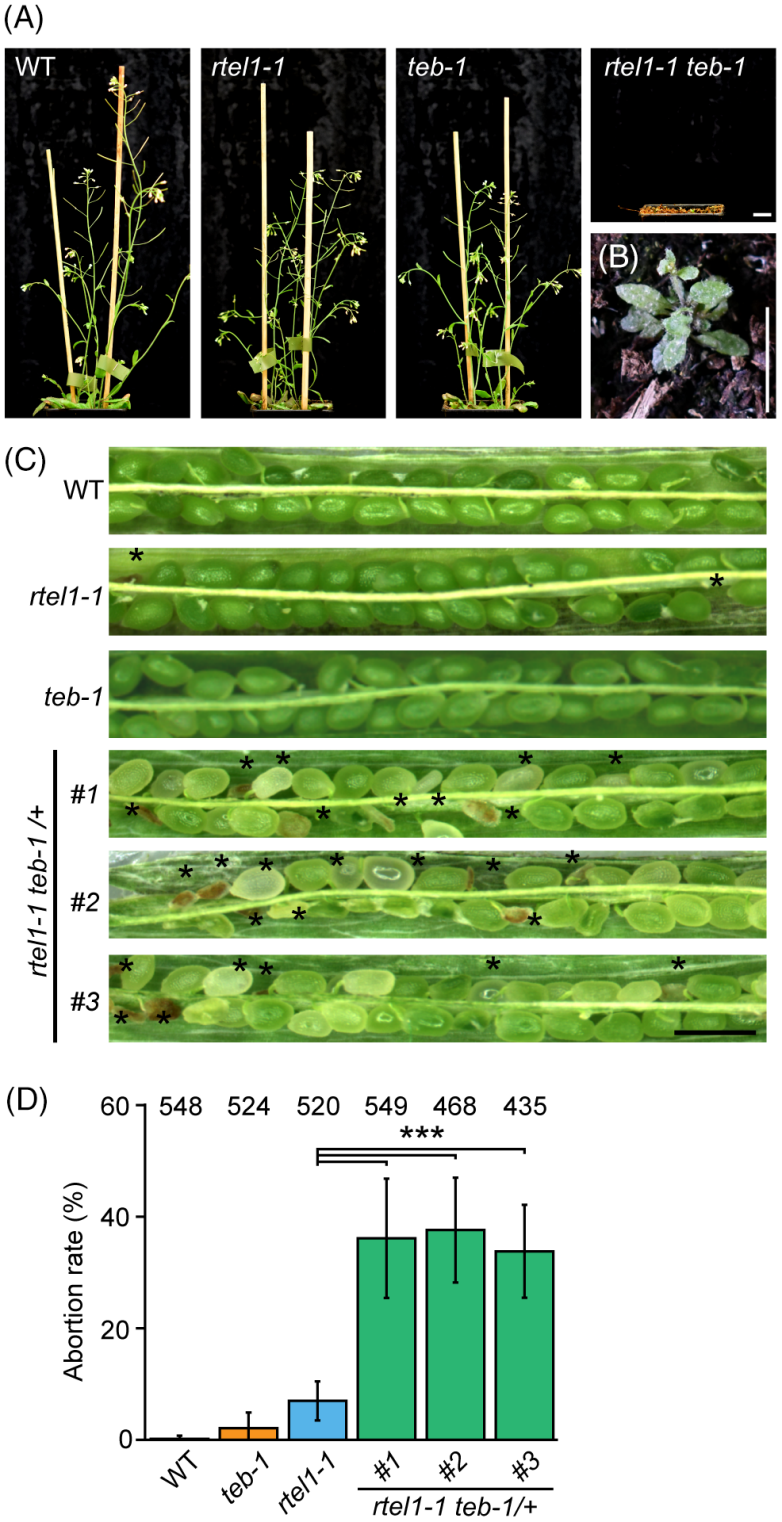
Simultaneous loss of *RTEL1* and *TEB* leads to embryo lethality. (A) Representative phenotypes of 6‐weeks‐old *rtel1‐1 teb‐1* double‐mutant, single‐mutant, and WT plants. Scale bar, 1 cm. (B) Detailed view of the *rtel1‐1 teb‐1* phenotype. (C) Representative content of siliques from self‐pollinated WT, *rtel1‐1* ^*−/−*^, *teb‐1* ^*−/−*^, and *rtel1‐1* ^*−/−*^ *teb‐1* ^*+/−*^ plants. Scale bar, 1 mm. (D) Percentage of aborted ovules/seeds of self‐fertilized WT and mutant plants. The numbers at the top of the bars correspond to the number of analyzed seeds. ***P < 0.00001 with chi‐square test. Error bars indicate the SD of the means of individually analyzed seeds. Source data are available in Table S5.

To explore the functional relationship in DPC repair between RTEL1 and TEB, we crossed *rtel1‐1* and *teb‐1* mutant plants. Surprisingly, we did not obtain any double homozygous mutants among approximately 80 analyzed F2 hybrid plants, indicating their possible lethality. To test this possibility, we analyzed the offspring of homozygous *rtel1‐1* and heterozygous *teb‐1* (*rtel1‐1* ^*−/−*^ *teb‐1* ^*+/−*^) plants, where 25% of plants were expected to be double homozygotes. Despite screening at least 400 plants from independent batches, including tiny and poorly growing plants, we identified only a single *rtel1‐1* ^*−/−*^ *teb‐1* ^*−/−*^ double homozygous mutant seedling (Figure 4A,B). The absence of homozygous double‐mutant plants coincided with a high number of 37% non‐germinating seeds (227/626 tested seeds), suggesting that simultaneous loss of RTEL1 and TEB causes seed lethality. To address this apparent discrepancy from the expected 25%, we harvested 10 green siliques from the central part of the main inflorescence stem of WT, *rtel1‐1*, *teb‐1*, and *rtel1‐1* ^*−/−*^ *teb‐1* ^*+/−*^ plants and analyzed numbers of normal and aborted seeds (Figure 4C; Table S5). WT control had only a small number of aborted seeds (0.2 ± 0.5%). The single *teb‐1* and *rtel1‐1* contained significantly increased numbers of aborted seeds (2.1 ± 2.8% and 7.0 ± 3.5%, respectively). The seeds of *rtel1‐1* ^*−/−*^ *teb‐1* ^*+/−*^ plants were analyzed from three independent individuals and showed 36.1 ± 10.7% (line 1), 37.6 ± 8.3% (line 2), and 33.8 ± 5.3% (line 3) of abortion, which was a significant increase compared to both WT and single mutants (Figure 4C,D; Table S5). Therefore, we conclude that RTEL1 and TEB function in parallel and synergistically during seed development, possibly by protecting the genome against replicative stress.

### SMC5/6 complex and RTEL1 function in parallel during plant development

To explore functional relationships of RTEL1/HZE2, TEB/HZE3, and previously characterized SMC6B/HZE1 factor (Dvořák Tomaštíková, Prochazkova, et al., 2023), we generated *rtel1‐1 smc6b‐1* and *teb‐1 smc6b‐1* homozygous double mutants.

**Figure 5:**
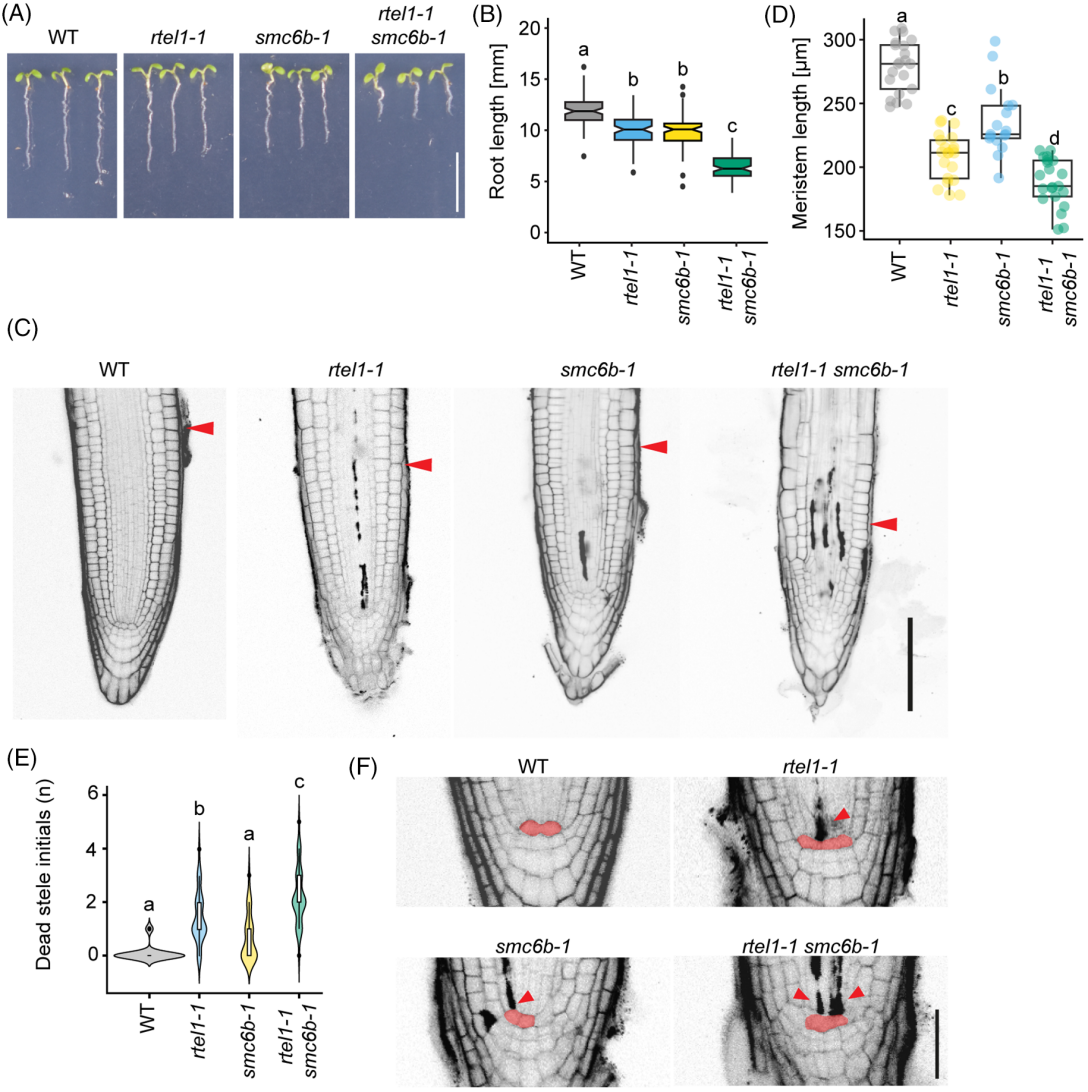
RTEL1 contributes to the mitigation of DNA damage independently of the SMC5/6 complex. (A) Root growth of 7‐day‐old WT, *rtel1‐1*, *smc6b‐1*, and *rtel1‐1 smc6b‐1*. Bar, 1 cm. (B) Quantification of the root length of plants shown in (A). Data are means ± SD from three biological replicates, each with a minimum of 20 seedlings. Different lowercase letters indicate significant differences (*P* < 0.05), according to one‐way anova followed by Tukey's test. Source data are available in Table S6. (C) Representative confocal microscopy images of plant roots shown in (A) stained with propidium iodide. Red arrowheads indicate the border of the meristematic and elongation zones of the root. Bar, 50 μm. (D) Average meristematic zone length in *rtel1‐1*, *smc6b‐1*, and *rtel1‐1 smc6b‐1* seedlings compared with the WT at 7 days after germination. Data are means ± SD from three biological replicates, each with a minimum of 20 seedlings. Different lowercase letters indicate significant differences (*P* < 0.05), according to one‐way anova followed by Tukey's test. Source data are available in Table S7. (E) Number of dead stele initials cells in 7‐day‐old WT, *rtel1‐1*, *smc6b‐1*, and *rtel1‐1 smc6b‐1*. Results are shown as violin plots with a boxplot, where the middle line represents the median, box boundaries signify the 25th and 75th percentiles, and whiskers the lowest and highest values. *N* = 2, *n* > 10. Different lowercase letters indicate significant differences (*P* < 0.05), according to one‐way anova followed by Tukey's test. Source data are available in Table S9. (F) Representative image of 7‐day‐old seedlings stained with propidium iodide. The QC is artificially colored in red, and dead stele initials are marked with arrowheads.

The root length of *rtel1‐1* (10.1 mm ± 0.2) and *smc6b‐1* (10.3 mm ± 0.9) plants was significantly shorter compared to WT plants (11.9 cm ± 0.0), and the roots of *rtel1‐1 smc6b‐1* plants (6.4 mm ± 0.6) were significantly shorter compared to all other genotypes under normal conditions (one‐way anova and Tukey HSD post hoc test, *P* < 0.05; note: the same test was used throughout this work; Figure 5A,B; Table S6). Microscopic analyses of root meristems after staining with PI revealed altered root meristem architecture in *rtel1‐1 smc6b‐1* relative to both single mutants (Figure 5C). Additionally, we observed that root meristems of the *rtel1‐1 smc6b‐1* plants were significantly shorter compared to the WT and single mutants (272.7 μm ± 25.4 in WT, 196.3 μm ± 24.2 in *rtel1‐1*, and 229.1 μm ± 25.3 in *smc6b‐1*, compared to 173.8 μm ± 19.5 in *rtel1‐1 smc6b‐1*) (Figure 5D; Table S7). This correlated with a significant decrease in the mean numbers of cortical meristem cells (26.0 ± 1.9 cells in WT, 17.3 ± 2.0 in *rtel1‐1*, 21.2 ± 2.3 in *smc6b‐1*, and 14.9 ± 1.6 in *rtel1‐1 smc6b‐1*) (Figure S9a; Table S8). The adult *rtel1‐1 smc6b‐1* plants were generally smaller than the respective single‐mutant and WT plants, but we did not observe any malformations or fertility‐related defects (Figure S9b,c). These data indicate the importance of RTEL1 and SMC6B in the early developmental phases associated with high cell division activity.

To investigate the spatial occurrence of cell death, WT, single‐, and double‐mutant plants were stained with PI. We noticed higher numbers of dead cells in the stem cell niche (SCN), especially in stele initials (Figure 5E,F). At 7 days after germination, no PI‐stained cells were observed in WT root meristems (0.09 ± 0.00 cells). Several stele initials were stained in *smc6b‐1* roots (0.73 ± 0.24, *P* > 0.05) and *rtel1‐1* roots (1.38 ± 0.41, *P* < 0.001). Significantly increased cell death in stele/vasculature initials was observed in *rtel1‐1 smc6b‐1* compared to all tested genotypes (2.23 ± 0.22, *P* < 0.001) (Figure 5E,F; Table S9). To investigate whether the high frequency observed in the double mutant might be a consequence of DNA replication stress, we quantified the duration of the S‐phase based on an incorporation of 5‐ethynyl‐20‐deoxyuridine (EdU) (Hayashi et al., 2013) and subsequent monitoring of EdU‐labeled cells as a function of time (Figure S9d). The average duration of S‐phase was 3.4 h in WT and 4.2 h in *smc6b‐1*. In contrast, the *rtel1‐1* single‐ and *rtel1‐1 smc6b‐1* double‐mutant plants had a significantly but similarly prolonged S‐phase duration of 7.6 and 7.3 h, respectively (Figure S9d). These results suggested that the increased amounts of dead cells in *rtel1‐1 smc6b‐1* double‐mutant plants were not caused by additional prolongation of S‐phase. In summary, altered root growth in *rtel1‐1 smc6b‐1* is associated with both increased cell death and partial loss of meristem identity as observed by severely affected root morphology (Figure 5A–D). Based on these experiments, we conclude that SMC5/6 complex and RTEL1 contribute to normal root growth and cell divisions via parallel pathways.

### TEB and RTEL1 function downstream of the SMC5/6 complex during the repair of ZEB‐induced DPCs

**Figure 6:**
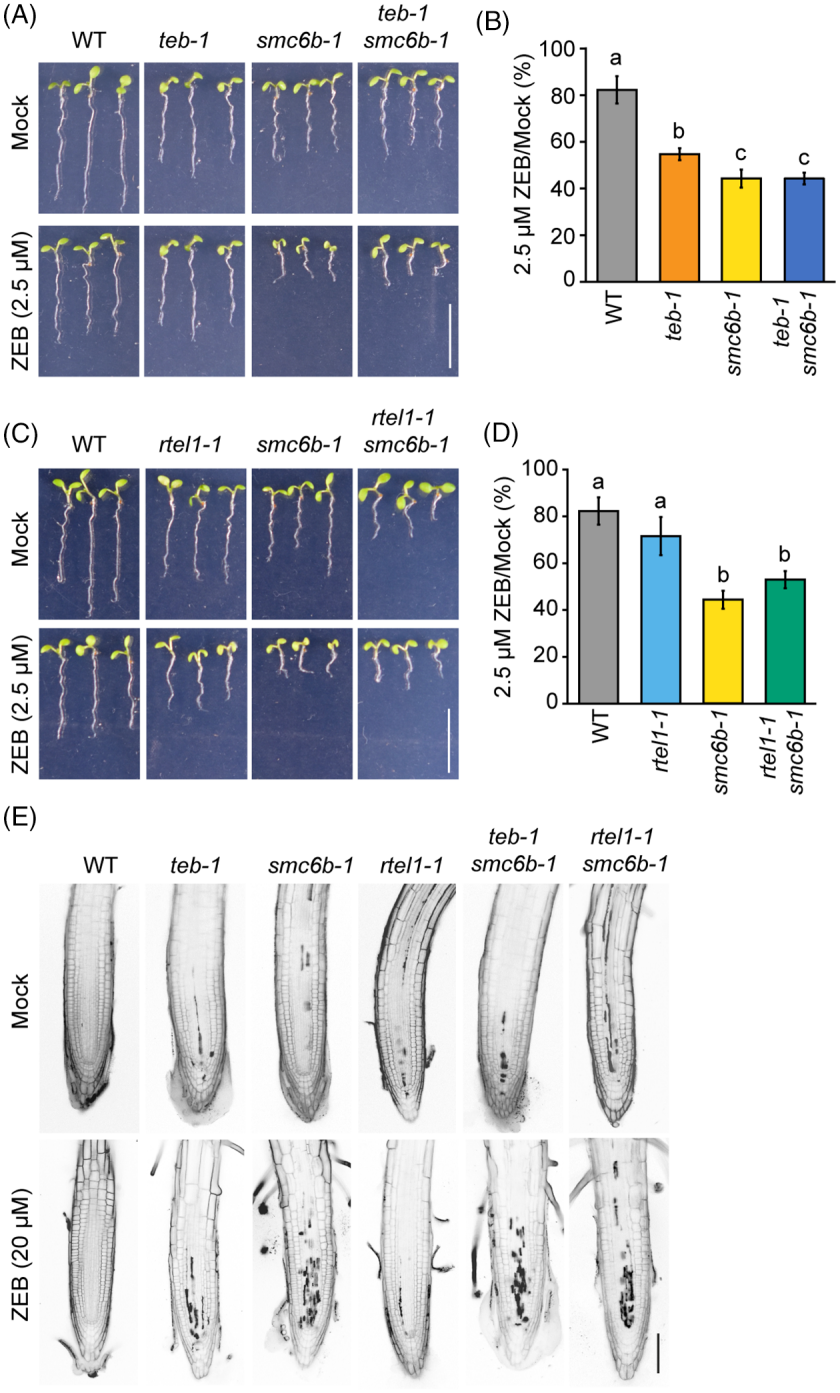
RTEL1 and TEB work downstream of SMC5/6 complex in the repair of ZEB‐induced DNA–protein crosslinks (DPCs). (A) Representative growth phenotypes on 0 (Mock) and 2.5 μm zebularine (ZEB). Scale bar, 1 cm. (B) Relative root length of seedlings in (A) under ZEB/mock conditions (% of ZEB/Mock). Data are means ± SD from three biological replicates, each with a minimum of 20 seedlings. Different lowercase letters indicate significant differences (*P* < 0.05), according to one‐way anova followed by Tukey's test. Source data are available in Table S11. (C) Representative growth phenotypes on 0 (Mock) and 2.5 μm ZEB. Scale bar, 1 cm. (D) Relative root length of seedlings in (C) under ZEB/mock conditions (% of ZEB/mock). Other details are as in (B). Source data are available in Table S12. (E) Representative confocal microscopy images of root tips stained with propidium iodide. Five‐day‐old seedlings were treated for 24 h with 20 μm ZEB prior to analysis. Scale bar, 100 μm.

Similar to *rtel1‐1 smc6b‐1*, we examined *teb‐1 smc6b‐1* phenotypes during normal development. The *teb‐1 smc6b‐1* plants developed normally, suggesting the absence or only minor genetic interaction under normal conditions (Figure 6A). Analyses of the root length after 7 days of growth on half‐strength Murashige and Skoog medium (½ MS) media without chemicals revealed shortening of the root length only to the extent of the respective single mutants (14.3 mm ± 1.0 in WT, 8.9 mm ± 1.4 in *teb‐1*, and 9.9 mm ± 0.7 in *smc6b‐1*, compared to 7.8 mm ± 0.6 in *teb‐1 smc6b‐1*; Figure S10a,b; Table S10). Accordingly, we did not observe an increased number of dead cells in the root meristematic zones (Figure S10c). No changes were observed at the later stages of plant development (Figure S10d). *TEB* mutants are susceptible to replicative stress (Nisa et al., 2021), and the fact that *teb‐1 smc6b‐1* plants do not have additive phenotype indicates that loss of *SMC6B* does not result in the induction of replicative damage per se.

Finally, we analyzed the sensitivity of the *teb‐1 smc6b‐1* and *rtel1‐1 smc6b‐1* double mutants to 2.5 μm ZEB (Figure 6A–D; Tables S11 and S12). Milder ZEB concentrations were used in this experiment to distinguish differences in highly sensitive *smc6b‐1* background. After 7 days of growth on mock or ZEB‐containing media, the roots of WT displayed an 18% length reduction (82.3% ± 5.8% of mock‐treated plants). For 2.5 μm ZEB‐treated *teb‐1* and *rtel1‐1* single mutants, only slight sensitivities were observed (54.7 ± 2.5% and 71.6 ± 8.1% of mock‐treated plants, respectively). The *smc6b‐1* and *teb‐1 smc6b‐1* showed both a 56% and *rtel1‐1 smc6b‐1* 47% reduction in root length (44.2 ± 3.8%, 44.3 ± 2.5%, and 53.0 ± 3.7% of mock‐treated plants, respectively). None of these sensitivities differed significantly compared to *smc6b‐1* (one‐way anova with Tukey post hoc test, *P* < 0.05). The PI staining of 20 μm ZEB‐treated *teb‐1 smc6b‐1* and *rtel1‐1 smc6b‐1* plants revealed a similar amount of dead cells as in the root meristematic zone of *smc6b‐1* plants (Figure 6E). Therefore, our genetic data suggest that RTEL1 and TEB function downstream of the SMC5/6 complex during the repair of ZEB‐induced DPCs.

## DISCUSSION

DNA–protein crosslinks encompass a highly diverse type of DNA damage, which contributes to the complexity and variety of the repair processes. Cells face the challenge of handling various proteins crosslinked to DNA by both internal and external factors at different stages of the cell cycle. The identification of specific DNA‐dependent proteases weak suppressor of *smt3* (Wss1) and SPARTAN in yeast and humans, respectively, responsible for cleaving to identify and characterize protein component of DPCs (Stingele et al., 2014; Vaz et al., 2016), has intensified research on DPC repair mechanisms. Despite the recent identification of a plant WSS1 homolog (Enderle et al., 2019; Stingele et al., 2015) and the discovery of several DPC repair pathways involving endonucleolytic cleavage, cleavage of the phosphodiesterase bond, or DPC SUMOylation (Dvořák Tomaštíková, Prochazkova, et al., 2023; Enderle et al., 2019; Hacker et al., 2022), DPC repair has received relatively little attention in plants. To extend our understanding of the DPC repair and tolerance mechanisms, we performed a forward‐directed genetic screen for sensitivity to cytidine analog zebularine that crosslinks MET1 to DNA (Prochazkova et al., 2022). In the ongoing follow‐up forward‐directed screen, we aimed to identify and characterize molecular factors required for the repair of zebularine‐induced DPCs (Dvořák Tomaštíková, Prochazkova, et al., 2023). In this study, we describe two mapped genes *HZE2* and *HZE3* as *RTEL1* and *TEB*, respectively. This connects HR and MMEJ pathways with the repair of MET1‐DPCs. Moreover, we show that although the role of SMC5/6 is mainly connected with its function in HR (Diaz et al., 2019; Watanabe et al., 2009), it also fulfills other functions in DPC repair, most likely through its SUMO E3 ligase activity (Dvořák Tomaštíková, Prochazkova, et al., 2023; Yang et al., 2021).

We identified three HZE2/RTEL1 missense variants, *hze2‐3*, *hze2‐4*, and *hze2‐5*, with mutations in the ATP‐binding pocket of the helicase domain, which belongs to the highly conserved SF2 helicase family. Mutations of SF2 family helicases were associated with severe genetic disorders due to genome instability in humans (Fujimoto et al., 2005). For example, the G47R mutation in xeroderma pigmentosum factor D (*Hs*XPD) is present in XP‐D patients, also featuring Cockayne syndrome manifested by dwarfism. We found a similar G90R mutation at the potentially homologous position within the *At*RTEL1 ATP‐binding motif I in *hze2‐3* (Figure 2A). Furthermore, the *hze2‐4* T94A (Figure S11) is localized at the position orthologous to the position of T49M mutation in human RTEL1, which is associated with familial pulmonary fibrosis (Kannengiesser et al., 2015). Based on structural modeling, we propose that like the human *Hs*XPD and *Hs*RTEL1 mutations, the Arabidopsis *hze2‐3* and *hze2‐4* variants disrupt the catalytic activity of the helicase domain crucial for its HR‐related function. Indeed, the *hze2‐3* mutant showed a defect in its protective role against spontaneous recombinogenic SDSA repair (Figure 2C). Interestingly, the *hze2‐1* mutation, which affects the binding of ssDNA has a significantly reduced frequency of SSA after ZEB treatment (Figure 2D). We speculate that during SSA, RTEL1 could help with the stabilization of displaced 3′ ssDNA strands (Vu et al., 2022).

We provide genetic evidence that RTEL1 works in parallel to the SMC5/6 complex during a standard replication regime with a low amount of DNA damage (Figure 5). RTEL1 plays a general role in promoting the progression of stalled forks (Campos et al., 2023), most likely through its function to unwind DNA ahead of a stalled replication fork to help overcome obstacles and to allow fork progression (Sparks et al., 2019). The SMC5/6 complex helps to stabilize challenged replication forks at DNA damage sites during replication stress (Palecek, 2018). In yeast cells, the SMC5/6 complex is recruited to the ssDNA‐dsDNA junctions, followed by SUMO‐mediated (NSE2‐dependent) recruitment of the DNA repair proteins (Tanasie et al., 2022). The SUMOylation of the protein adducts stimulates proteasomal degradation outside replication (Liu et al., 2021). Therefore, we hypothesize that, to compensate for the lack of RTEL1 and the presence of toxic recombination intermediates that occur naturally during DNA replication (Lambert et al., 2007), the SMC5/6 complex stabilizes stalled replication forks and promotes SUMO‐dependent repair. We assume that *rtel1‐1 smc6b‐1* had naturally increased levels of DNA damage that were sufficient to trigger cell death in the SCN observed in our study (Figure 5).

In cancer cells with impaired HR, TEB/POLQ plays a crucial role in the DSB repair (Ceccaldi et al., 2015). We have identified TEB as an important factor in the repair of replication stress‐coupled DNA damage in the absence of RTEL1 (Figure 4). We propose that, as in the mammalian cells, TEB becomes the primary repair factor of the stalled replication forks in the absence of functional HR in Arabidopsis. Importantly, both MMEJ and HR require resected ends for successful repair (Ceccaldi et al., 2016). In the absence of both HR and MMEJ (as in the *rtel1‐1 teb‐1* mutant), the resected ends cannot be further processed (Schrempf et al., 2021). Additionally, TEB participates in the repair of the DNA damage caused by ZEB in the same pathway as the SMC5/6 complex (Figure 6). Based on our previous genetic data (Dvořák Tomaštíková, Prochazkova, et al., 2023), we hypothesize that the SUMO modification mediated by the SMC5/6 complex is essential for the guidance of the repair machinery during this process. Speculatively, the function of Arabidopsis SMC5/6 complex function during replication could be ensured by SMC6A. Simultaneous loss of function from SMC6A and SMC6B is embryo lethal (Zou et al., 2021).

**Figure 7:**
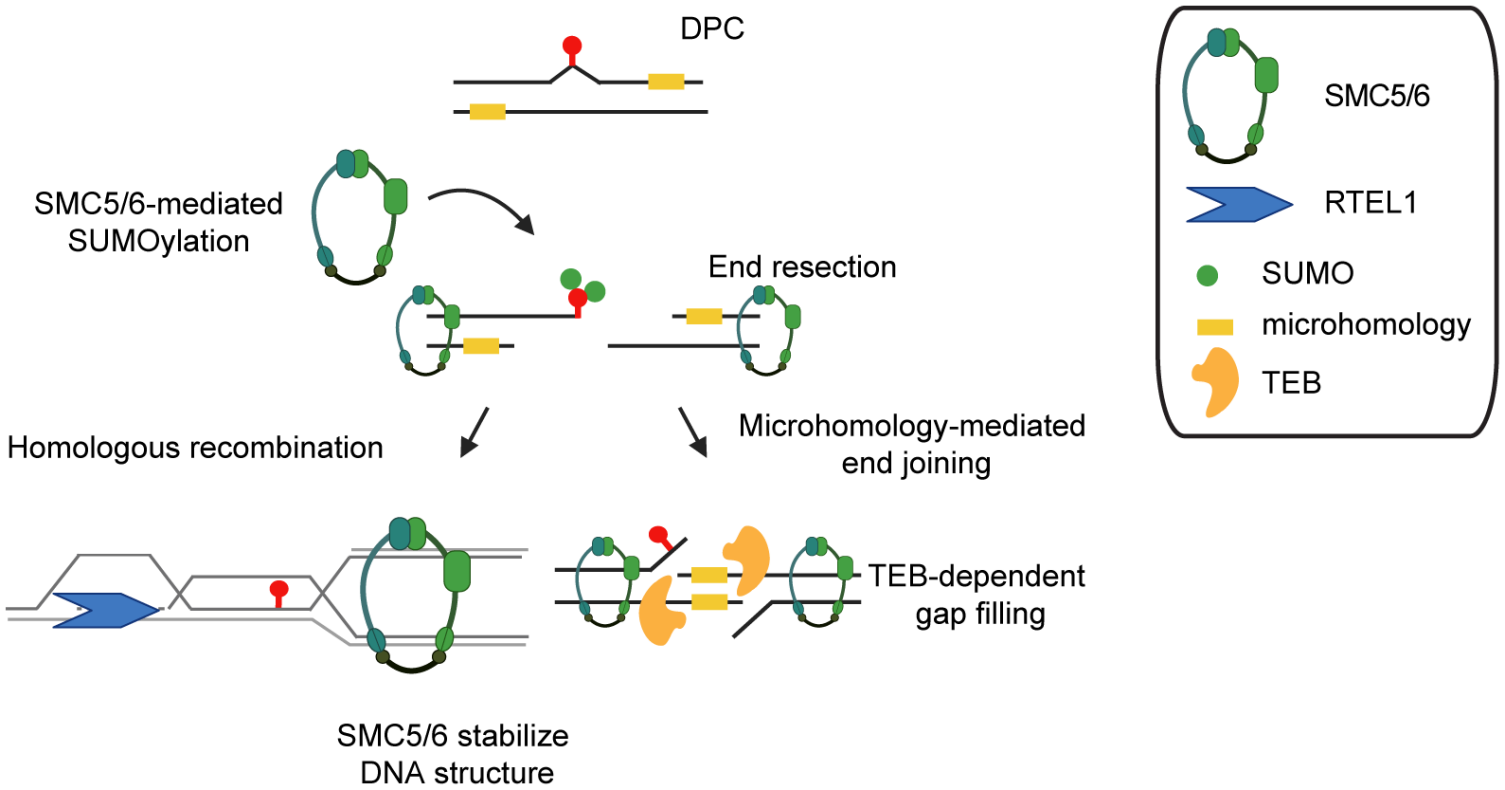
Working model of the role of the SMC5/6, RTEL1, and TEB in the maintenance of plant genome integrity. After recognition of DNA–protein crosslink (DPC) induced by zebularine (red lollipop), DNA ends that are presumably resected are subsequently stabilized by the SMC5/6. Additionally, the SMC5/6 complex might stimulate SUMO‐dependent degradation of MET1‐DPC. Subsequently, parallel repair pathways contribute to the repair. The DPC‐engaged DNA will be repaired either via the synthesis‐dependent strand annealing (SDSA) type of HR in RTEL1‐dependent manner or by MMEJ pathway represented (among other factors not shown here) by TEBICHI.

In conclusion, we strengthen the evidence that several DNA damage repair pathways collaborate to repair bulky DNA damage, such as DPCs in plants. We propose differential roles of RTEL1, TEB, and SMC5/6 complex in response to naturally occurring DNA replication barriers and toxic DPCs. Our genetic data suggest that under normal conditions, RTEL1 works in parallel to TEB, presumably to restart stalled replication forks. SMC5/6 might contribute to the stabilization of stalled replication forks in both of these pathways. During the repair of ZEB‐induced DPCs, the SMC5/6 SUMOylation can stimulate SUMO‐dependent degradation followed by microhomology‐directed repair by TEB or HR (Figure 7). Genetic interactions of the SMC5/6 complex with several DPC repair pathways suggest that it is a major integrator of the repair processes. Altogether, our study demonstrates the growing complexity of DPC repair in plants.

## MATERIALS AND METHODS

### Plant materials

*Arabidopsis thaliana* WT and mutants in Col‐0 background (unless stated otherwise) were used in this study: *smc6b‐1* (SALK_123114C), *rtel1‐1* (SALK_113285) (Recker et al., 2014), and *teb‐1* (Inagaki et al., 2006). The double mutants were generated by crosses of homozygous single mutants and were identified in the F2 generation by PCR‐based genotyping. The oligonucleotides used for genotyping are listed in Table S13. Plants were cultivated in an air‐conditioned phytochamber with a long day regime (16 h light, 150 μmol m^−2^ sec^−1^, 21°C, 8 h dark, 19°C; light provided by fluorescent tube MASTER TL‐D 18 W/840; Philips). For the drug sensitivity assays, seeds were surface sterilized using 8% sodium hypochlorite solution for 6 min, followed by three washes in sterile H_2_O, stratified for 2 days at 4°C in dark, and evenly distributed on plates with ½ MS with 0.6% agar and with or without the addition of DNA–protein crosslinking chemicals, depending on the experimental setup.

### Forward‐directed genetic screen and MBS

The HZE genetic screen was performed as described (Dvořák Tomaštíková, Prochazkova, et al., 2023). Briefly, the W35 reporter line (Willing et al., 2016) was mutagenized with 0.2% ethyl methanesulfonate. The M1 generation was grown as pools of 100 plants (batches) until maturity. M2 seeds of all plants in a batch were collected and screened for ZEB‐sensitive mutant candidates. Internal zebularine‐sensitive *smc6b‐1* and resistant WT controls were included on each plate. Each M2 primary candidate was further analyzed by phenotyping at the M3 generation on the ½ MS media without and with 20 μm zebularine (approximately 30 plants per experimental point). The candidates selected for mapping were back‐crossed to the non‐mutagenized WT, and their BCF2 population was screened on 20 μm zebularine‐containing ½ MS media. About 75–150 zebularine‐sensitive plants were collected and pooled, and their genomic DNA was isolated using the NucleoSpin Plant II kit (Macherey‐Nagel, Dueren, Germany). Genomic DNA was sent for commercial sequencing (Novogene Ltd., Cambridge, UK), using PE150 mode on the Novaseq platform to approximately 50× coverage. The reads were mapped to the *A. thaliana* reference genome (Araport11) with bowtie2 in default settings (Langmead & Salzberg, 2012). Read sorting, SNP calling, and filtering were done using tools from the MiModD tool set (Moos et al., 2014) and annotated with the snpEff tool (Cingolani et al., 2012).

### Root length assays and phenotypic analyses of mutant plants

Stratified, surface‐sterilized seeds were evenly sown on square culture plates with ½ MS, 0.8% agar, and cultivated horizontally for 7 days. Subsequently, the plants were carefully pulled from agar with tweezers and stretched on agar plates. Seedlings were photographed with a D90 digital camera (Nikon), and the primary root length was measured using the ImageJ plugin SmartRoot (Lobet et al., 2011). Details were photographed using an SZX16 binocular microscope equipped with Regita 1300 QImaging camera and QCapture x64 software (both Olympus).

For DNA damage sensitivity assays of single mutants, plants were germinated on ½ MS, 0.8% agar supplemented with chemicals: 20 μm zebularine (Z4775; Sigma‐Aldrich, Praha, Czech Republic), 20 nm CPT (C9911; Sigma‐Aldrich, Praha, Czech Republic), and 10 μm ICRF‐187 (D1446; Sigma‐Aldrich, Praha, Czech Republic). Sensitivity to each chemical treatment in individual replicates was determined by calculating: mean (treatment)/mean (mock). The experiment was performed in three biological replicates, each with at least 20 plants/replicate. The means of the three replicates are depicted. The normality of data distribution was tested by the Shapiro–Wilk test. Statistical significance was tested with one‐way anova with post hoc Tukey HSD in R (RStudio Team, 2020).

Drug sensitivity assays were performed as described (Dorn & Puchta, 2020). Stratified, surface‐sterilized seeds were sown on culture plates with ½ MS, 0.6% agar, and cultivated for 7 days. Subsequently, 10 plantlets of each genotype were transferred to a six‐well culture plate containing 5 ml of liquid ½ MS media (untreated control) or 4 ml of liquid ½ MS media (treated samples) per well under sterile conditions. The next day, 1 ml of genotoxin solution diluted in liquid ½ MS was added to obtain the desired final concentration. The fresh weight was measured after 13 days of exposure. The relative fresh weight was determined by comparison of fresh weight between treated and untreated samples for each genotype and concentration. The experiment was performed in three biological replicates, and the means of the three replicates are depicted. Statistical significance was tested with one‐way anova with post hoc Tukey HSD in R (RStudio Team, 2020).

### Cell death analyses in roots

Seeds were grown on vertically positioned plates with ½ MS, 0.6% agar for 5 days, and then transferred to liquid ½ MS media without (mock), with 20 μm zebularine, 20 nm CPT, or 10 μm ICRF‐187 for 24 h. Afterward, the seedlings were placed in 10 mg ml^−1^ of PI solution (Sigma) on slides, immediately analyzed, and photographed using Leica confocal microscope TCS SP8 (Leica, Wetzlar, Germany) and HC PL APO CS2 20×/0.75 DRY objective equipped by Leica LAS‐X software with Leica lightning module laser scanning confocal microscope (Leica). The pattern was checked in at least 10 individual plants per treatment.

### Analyses of HDR

The HDR assays were performed as described (Vladejić et al., 2022). Briefly, *B11*, *B11 hze2‐1*, B11 *hze2‐3*, *IC9C*, *IC9C hze2‐1*, and *IC9C hze2‐3* plants were grown on ½ MS medium without (mock) or with 2.5 μm ZEB under sterile conditions. Ten‐day‐old seedlings were histochemically stained using GUS staining buffer (0.1 m sodium phosphate buffer [pH 7.0], 0.05% Triton X‐100, 0.1% X‐Glc sodium salt (Thermo Fisher, Brno, Czech Republic) containing 1 mm K_3_[Fe(CN)_6_], and 1 mm K_4_[Fe(CN)_6_]) for overnight at 37°C, as described (Dvořák Tomaštíková, Yang, et al., 2023). Subsequently, the GUS staining solution was removed, and plants were cleared by overnight incubation in 70% ethanol (v/v) at 37°C. Ethanol was changed three times, and after the last change, plants were left overnight at 4°C. Plants were transferred to a Petri dish containing ethanol and examined using a stereo‐microscope (Olympus SZX16) for HR events identified as blue‐stained cells or areas. The means of the three replicates are depicted. Statistical significance was tested with the Kruskal–Wallis *H*‐test with the post hoc Conover–Iman test of multiple comparisons with the Benjamini–Hochberg procedure in R (RStudio Team, 2020).

### EdU pulse labeling

The S phase length analyses were performed as described (Hayashi et al., 2013) with minor modifications. Plants were grown on solid ½ MS media for 5 days and transferred to liquid ½ MS media supplemented with 10 μm EdU (Thermo Fisher, Brno, Czech Republic). Samples were collected at 3, 6, 9, and 12 h, and fixed in 4% formaldehyde in 1× PBS, 0.5% Triton X‐100, and subjected to the Click‐iT reaction in 1× PBS, 4 mm CuSO4, 40 mm sodium ascorbate, and 5 μm AF488 azide. At least five roots of each genotype and time point were analyzed and photographed using Leica confocal microscope TCS SP8 (Leica) and HC PL APO CS2 20×/0.75 DRY objective equipped by Leica LAS‐X software with Leica lightning module laser scanning confocal microscope (Leica). Maximal projections of *Z*‐stacks (2.74 μm total width) comprising five layers were analyzed. Excitation was done using excitation laser 405 nm (DAPI) and 499 nm (Alexa488) with shutter intensity not exceeding 8%, and signal was detected using HyD detectors (410–494 nm DAPI and 504–690 nm Alexa488). EdU‐labeled nuclei in the root meristematic zone were counted, and the percentage of EdU‐positive nuclei were plotted as a function of time, as described in Eekhout et al. (2021). The percentage of EdU‐positive nuclei increases linearly with time and follows an equation that can be written as *y* = *at* + *b*, where *y* is the percentage of EdU‐positive nuclei and *t* is time. The S phase length is *b*/*a*.

### *In silico* protein analysis

The *At*RTEL1 AlphaFold structural model from the EMBL‐EBI database was used (AF‐F4HQE2‐F1; Varadi et al., 2022). Using the 6FWS crystal structure of the bacterial DinG helicase (Cheng & Wigley, 2018), ATP and ssDNA molecules were manually docked to the *At*RTEL1 ATP‐binding pocket and DNA‐binding groove, respectively. The structures were aligned and visualized using the PyMOL software (Schrodinger, Inc., USA).

### ACCESSION NUMBERS

Sequence data of the genes used in this article can be found at TAIR with the following accession numbers: *SMC6B* (AT5G61460), *RTEL1* (AT1G79950), and *TEB* (AT4G32700).

## AUTHOR CONTRIBUTIONS

AP and EDT designed the research; EDT, JV, VS, KK, and ZP performed the research; EDT, JV, JJP, VS, KK, and ZP analyzed data; EDT and AP wrote the paper. All authors read and approved the manuscript.

## CONFLICT OF INTEREST STATEMENT

The authors declare no conflicts of interest.

## Supporting information


**Figure S1.** Mapping‐by‐sequencing (MBS) of *hze2‐1* mutation.
**Figure S2.** Complementation crosses of selected *hze2* and *hze3* candidates.
**Figure S3.** Mapping‐by‐sequencing (MBS) of *hze2‐2* mutation.
**Figure S4.** Mapping‐by‐sequencing (MBS) of *hze2‐3* mutation.
**Figure S5.** Mapping‐by‐sequencing (MBS) of *hze2‐4* mutation.
**Figure S6.** Mapping‐by‐sequencing (MBS) of *hze2‐5* mutation.
**Figure S7.** Sensitivity of *hze2/rtel1* to type 2 and 4 DNA‐protein crosslinking agents.
**Figure S8.** Mapping‐by‐sequencing (MBS) of *hze3‐1* mutation.
**Figure S9.** Phenotypic analysis of *rtel1‐1*, *smc6b‐1*, and *rtel1‐1 smc6b‐1* mutants under normal conditions.
**Figure S10.** Phenotypic analysis of *teb‐1*, *smc6b‐1*, and *teb‐1 smc6b‐1* mutants under normal conditions.
**Figure S11.** Structural analysis of *hze2‐4*.
**Table S1.** Source data for the statistical analyses to support Figure 1(B).
**Table S2.** Source data for the statistical analyses to support Figure 7.
**Table S3.** Source data for the statistical analyses to support Figure 2.
**Table S4.** Source data for the statistical analyses to support Figure 3.
**Table S5.** Source data to support Figure 4.
**Table S6.** Source data to support Figure 5(B).
**Table S7.** Source data to support Figure 5(D).
**Table S8.** Source data to support Figure 9(A).
**Table S9.** Source data to support Figure 5(E).
**Table S10.** Source data to support Figure 10(B).
**Table S11.** Source data to support Figure 6(B).
**Table S12.** Source data to support Figure 6(D).
**Table S13.** Primers used in this study.
