# Supplementary material for: The interplay of homology‐directed repair pathways in the repair of zebularine‐induced DNA–protein crosslinks in Arabidopsis
Source: Plant J. 2024 Jun 2;119(3):1418–32. doi: 10.1111/tpj.16863 (PMC13087486; doi:10.1111/tpj.16863)
Supplement: Supplementary file 1 — Figure S1. Mapping‐by‐sequencing (MBS) of hze2‐1 mutation. Figure S2. Complementation crosses of selected hze2 and hze3 candidates. Figure S3. Mapping‐by‐sequencing (MBS) of hze2‐2 mutation. Figure S4. Mapping‐by‐sequencing (MBS) of hze2‐3 mutation. Figure S5. Mapping‐by‐sequencing (MBS) of hze2‐4 mutation. Figure S6. Mapping‐by‐sequencing (MBS) of hze2‐5 mutation. Figure S7. Sensitivity of hze2/rtel1 to type 2 and 4 DNA‐protein crosslinking agents. Figure S8. Mapping‐by‐sequencing (MBS) of hze3‐1 mutation. Figure S9. Phenotypic analysis of rtel1‐1, smc6b‐1, and rtel1‐1 smc6b‐1 mutants under normal conditions. Figure S10. Phenotypic analysis of teb‐1, smc6b‐1, and teb‐1 smc6b‐1 mutants under normal conditions. Figure S11. Structural analysis of hze2‐4. Table S1. Source data for the statistical analyses to support Figure 1(B). Table S2. Source data for the statistical analyses to support Figure 7. Table S3. Source data for the statistical analyses to support Figure 2. Table S4. Source data for the statistical analyses to support Figure 3. Table S5. Source data to support Figure 4. Table S6. Source data to support Figure 5(B). Table S7. Source data to support Figure 5(D). Table S8. Source data to support Figure 9(A). Table S9. Source data to support Figure 5(E). Table S10. Source data to support Figure 10(B). Table S11. Source data to support Figure 6(B). Table S12. Source data to support Figure 6(D). Table S13. Primers used in this study. [file TPJ-119-1418-s001.docx]

**SUPPLEMENTAL FIGURES**


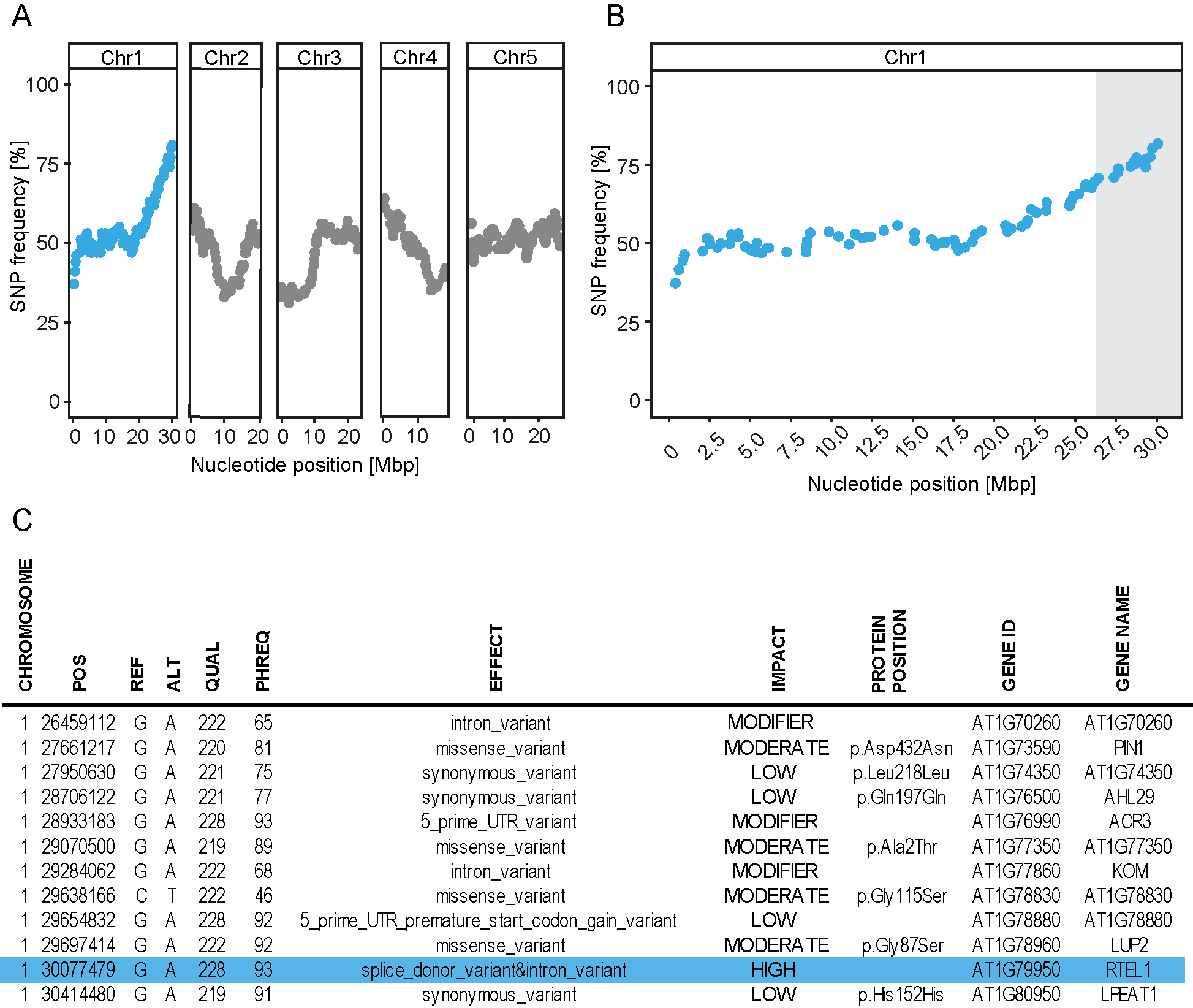


**Supplemental Figure 1 (related to Figure 1): Mapping-by-sequencing (MBS) of *hze2-1* mutation.** A, The SNP frequency plot based on the sequencing of gDNA from ~100 zebularine-sensitive BCF2 plants. Only SNPs with a QUAL score ≥ 100 were used for the plot calculation. Individual points represent the average allele frequency of eleven consecutive mutations (sliding window 11). B, Close-up view of the SNP frequency plot at the position containing candidate gene on chromosome 1. The main candidate region on chromosome 1 bottom arm is underlined by a grey background. C, Table of the candidate SNPs in the candidate region. The blue background highlights the causal SNP at the *RTEL1* (AT1G79950). SNP caused a missense mutation and was 93 % (PHREQ) in the population of sensitive plants selected for the mapping. POS = position on the chromosome, REF = nucleotide in reference sequence; ALT = altered nucleotide, QUAL = PHRED quality score.


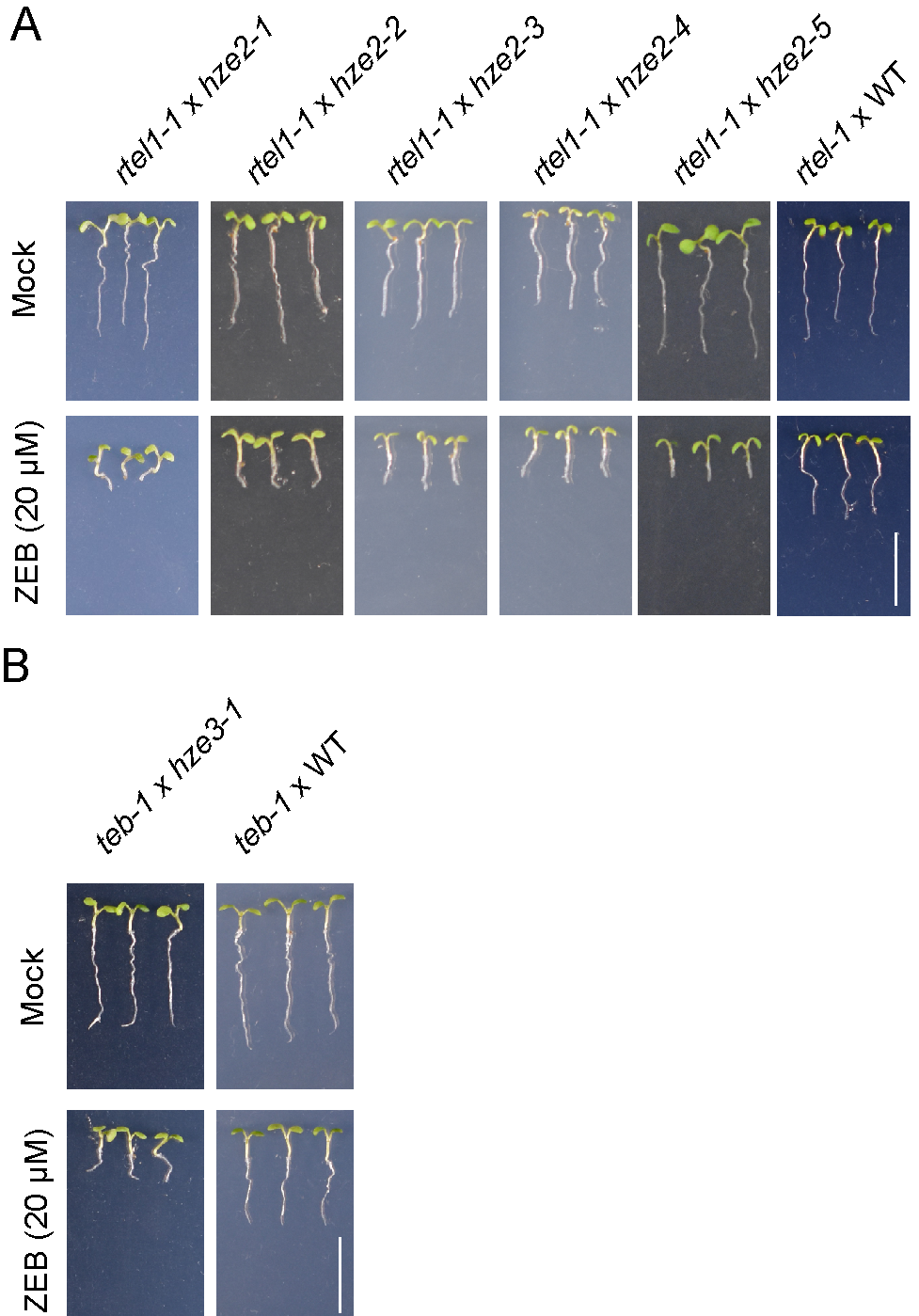
**Supplemental Figure 2 (related to Figures 1 and 3): Complementation crosses of selected *hze2* and *hze3* candidates.** A, Representative growth phenotypes of F1 hybrid seedlings generated from complementation crosses of *hze2* with *rtel1-1*. See Figure 1A for the phenotype of the controls. Bar, 1 cm. B, Representative growth phenotypes of F1 hybrid seedlings generated from complementation crosses of *hze3* with *teb-1*. See Figure 3A for the phenotype of the controls. Bar, 1 cm.


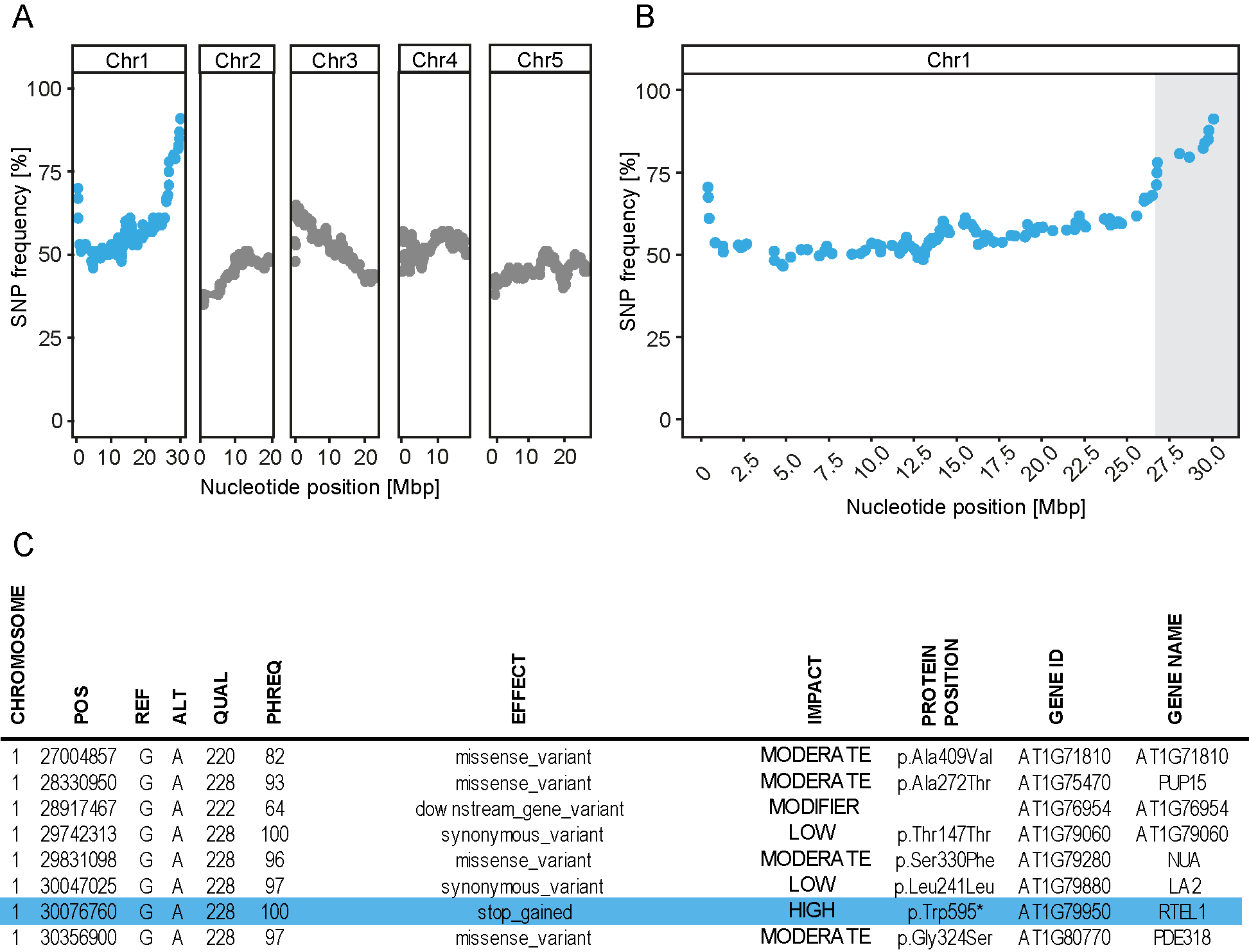


**Supplemental Figure 3 (related to Figure 1): Mapping-by-sequencing (MBS) of *hze2-2* mutation.** A, The SNP frequency plot based on the sequencing of gDNA from ~100 zebularine-sensitive BCF2 plants. Other details are as in Supplemental Figure 1.


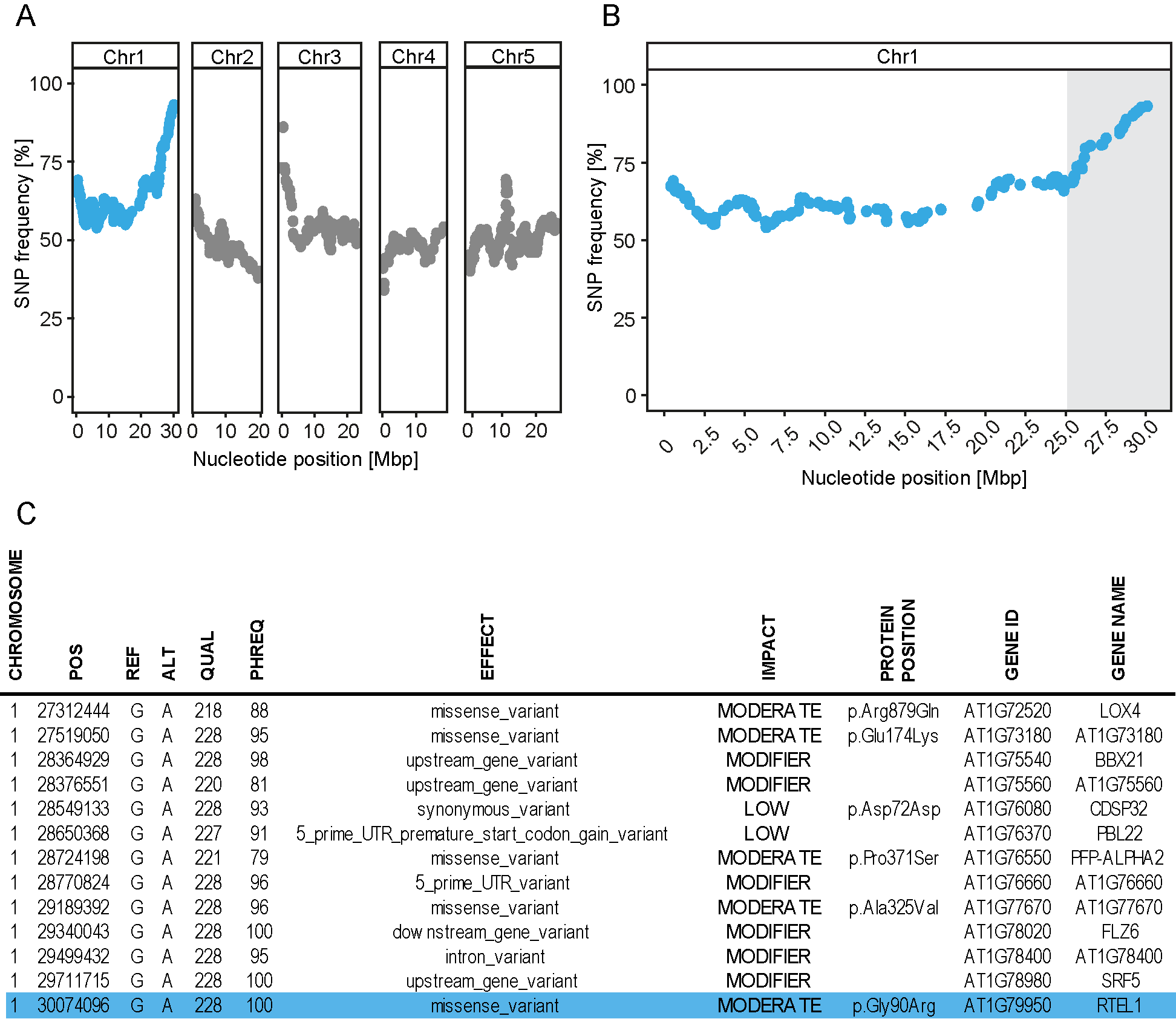


**Supplemental Figure 4 (related to Figure 1): Mapping-by-sequencing (MBS) of *hze2-3* mutation.** A, The SNP frequency plot based on the sequencing of gDNA from ~100 zebularine-sensitive BCF2 plants. Other details are as in Supplemental Figure 1.

**
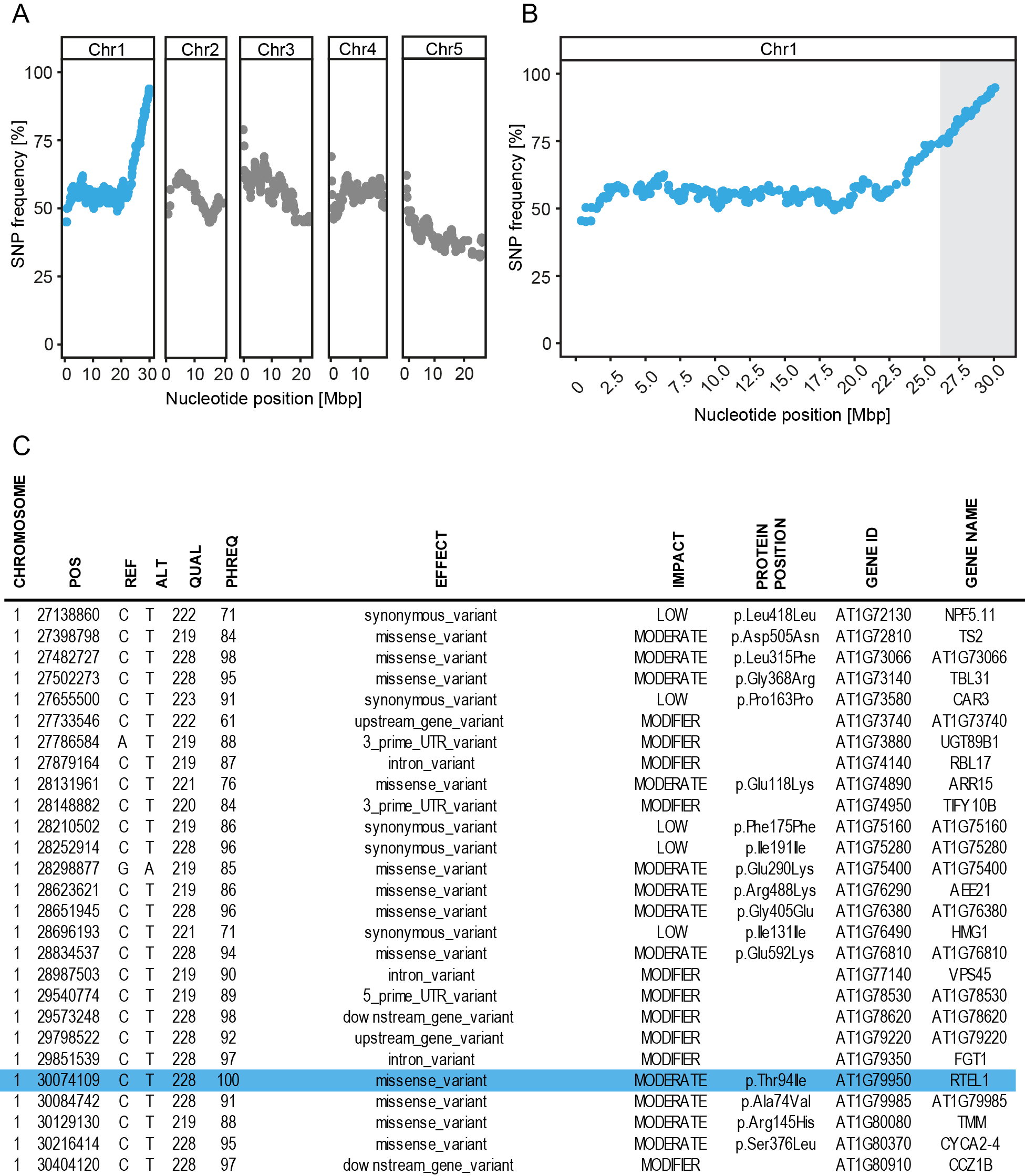
**

**Supplemental Figure 5 (related to Figure 1): Mapping-by-sequencing (MBS) of *hze2-4* mutation.** A, The SNP frequency plot based on the sequencing of gDNA from ~100 zebularine-sensitive BCF2 plants. Other details are as in Supplemental Figure 1.


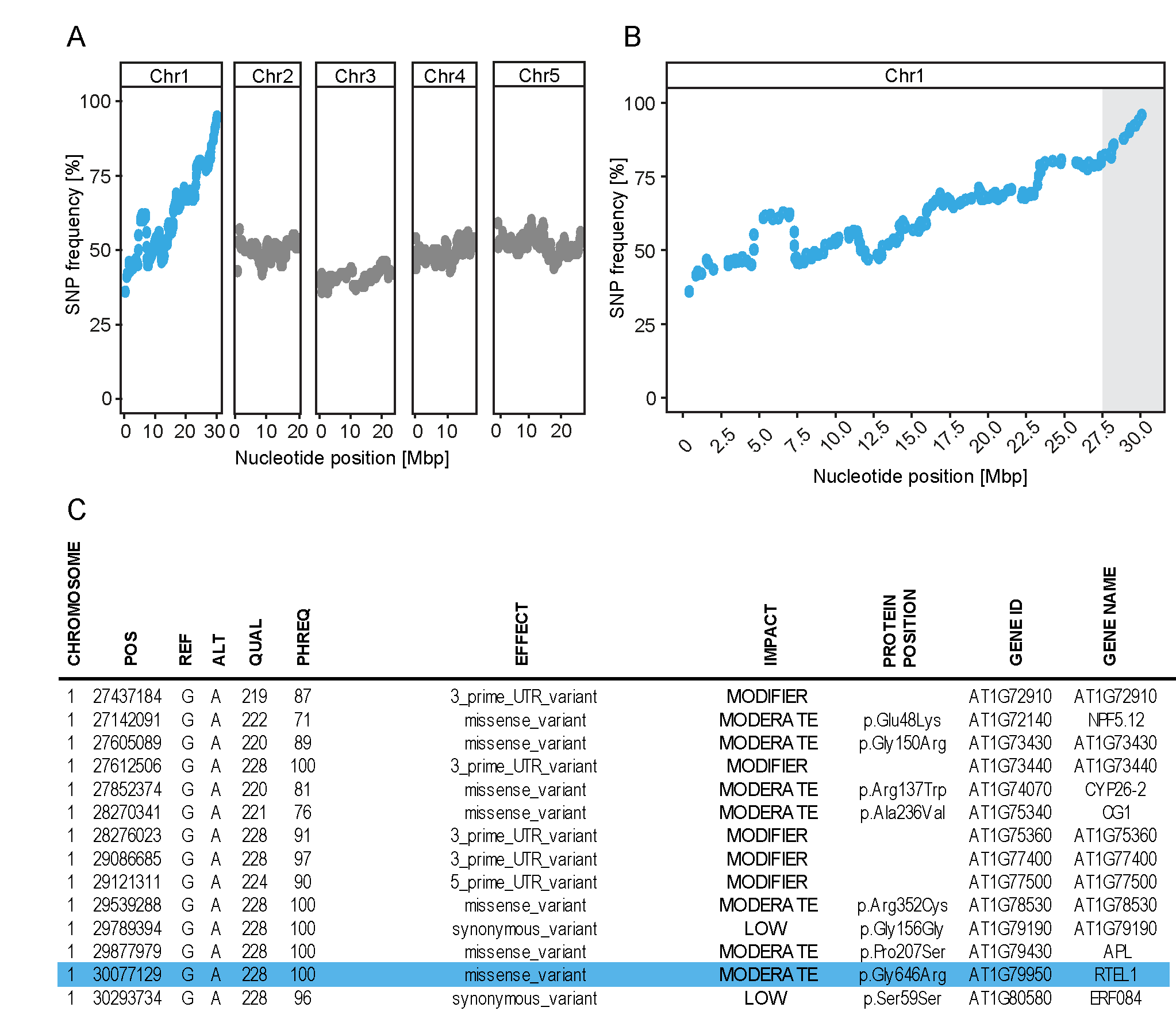


**Supplemental Figure 6 (related to Figure 1): Mapping-by-sequencing (MBS) of *hze2-5* mutation.** A, The SNP frequency plot based on the sequencing of gDNA from ~100 zebularine-sensitive BCF2 plants. Other details are as in Supplemental Figure 1.


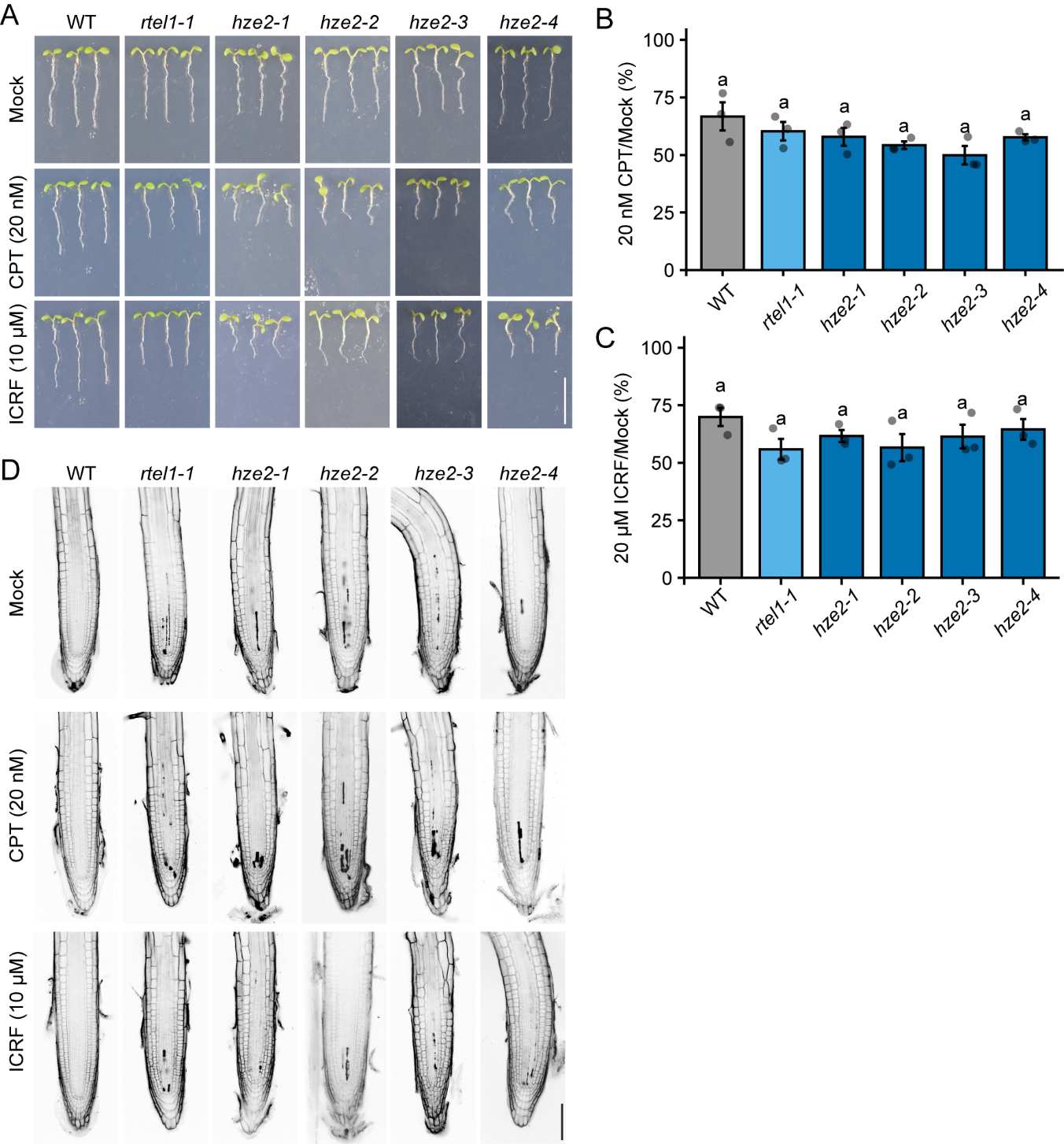


**Supplemental Figure 7 (related to Figure 1): Sensitivity of *hze2/rtel1* to type 2 and 4 DNA-protein crosslinking agents.** A, Representative growth phenotypes of seedlings from wild-type (WT), *rtel1-1* and *hze2* on 0 (Mock), 20 nM camptothecin (CPT), and 10 μM dexrazoxane (ICRF). Scale bar, 1 cm. B, Relative root length of seedlings in (A) under camptothecin/mock conditions. Data are means ± SD from three biological replicates, each with a minimum of 20 seedlings. Different lowercase letters indicate significant differences (*P*<0.05), according to one-way ANOVA followed by Tukey’s test. Source data for statistical analyses are available in Supplemental Supplemental Table 2. Note: The original experiment was split between Figures 1A, and Supplementary Figure 7A. Therefore, these figures show identical images and data for the controls. C, Relative root length of seedlings in (A) under ICRF/mock conditions. Data are means ± SD from three biological replicates, each with a minimum of 20 seedlings. Different lowercase letters indicate significant differences (*P*<0.05), according to one-way ANOVA followed by Tukey’s test. Source data for statistical analyses are available in Supplemental Table2. D, Representative confocal microscopy images of root tips stained with propidium iodide, which indicates dead cells (dark sectors). Five-day-old seedlings were treated with 20 nM CPT and 10 μM ICRF for 24 h prior to analysis. Scale bar, 100 μm.


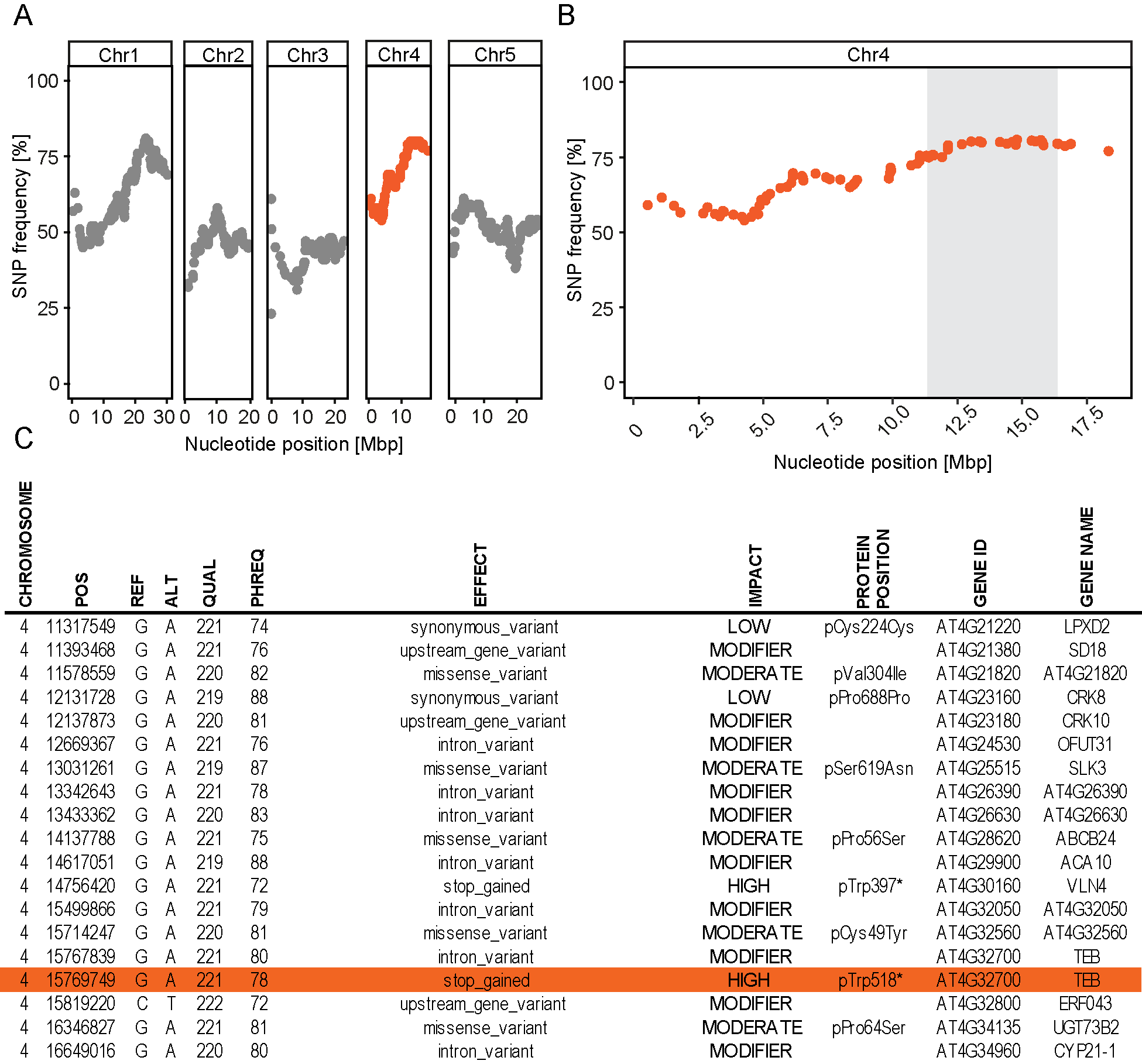


**Supplemental Figure 8 (related to Figure 3): Mapping-by-sequencing (MBS) of *hze3-1* mutation.** A, The SNP frequency plot based on the sequencing of gDNA from ~100 zebularine-sensitive BCF2 plants. Only SNPs with a QUAL score ≥ 100 were used for the plot calculation. B, Close-up view of the SNP frequency plot at the position containing candidate gene on chromosome 4. C, Table of the candidate SNPs in the candidate region. The orange background highlights the causal SNP at the TEB1 (AT4G32700). SNP caused a premature stop codon and was 78% (PHREQ) in the population of sensitive plants selected for the mapping. Other details are as in Supplemental Figure 1.

**
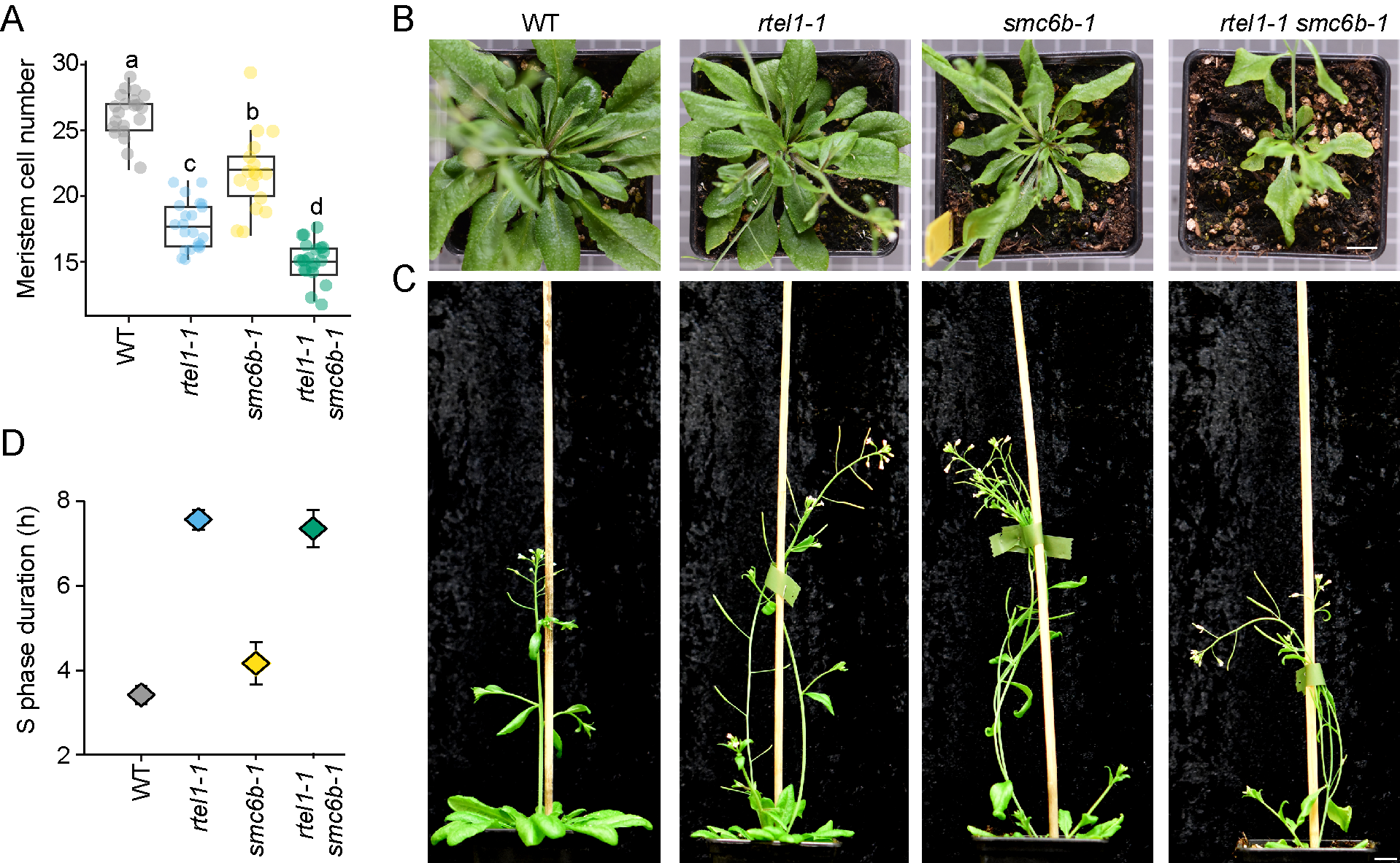
**

**Supplemental Figure 9 (related to Figure 5): Phenotypic analysis of *rtel1-1*, *smc6b-1,* and *rtel1-1 smc6b-1* mutants under normal conditions.** A, Meristem cell number in *rtel1-1*, *smc6b-1,* and *rtel1-1 smc6b-1* seedlings compared with the WT at 7 days after germination. Data are means ± SD from three biological replicates, each with a minimum of 20 seedlings. Different lowercase letters indicate significant differences (*P*<0.05), according to one-way ANOVA followed by Tukey’s test. Source data are available in Supplemental Table8. B, C, Representative phenotypes of six-week-old plants grown on soil from a top view (B) and side view (C). Scale bar, 4 cm. D, S phase duration was measured as the time course of EdU-labelled nuclei. Data represent the mean of 5 technical replicates ± 95% confidence intervals.


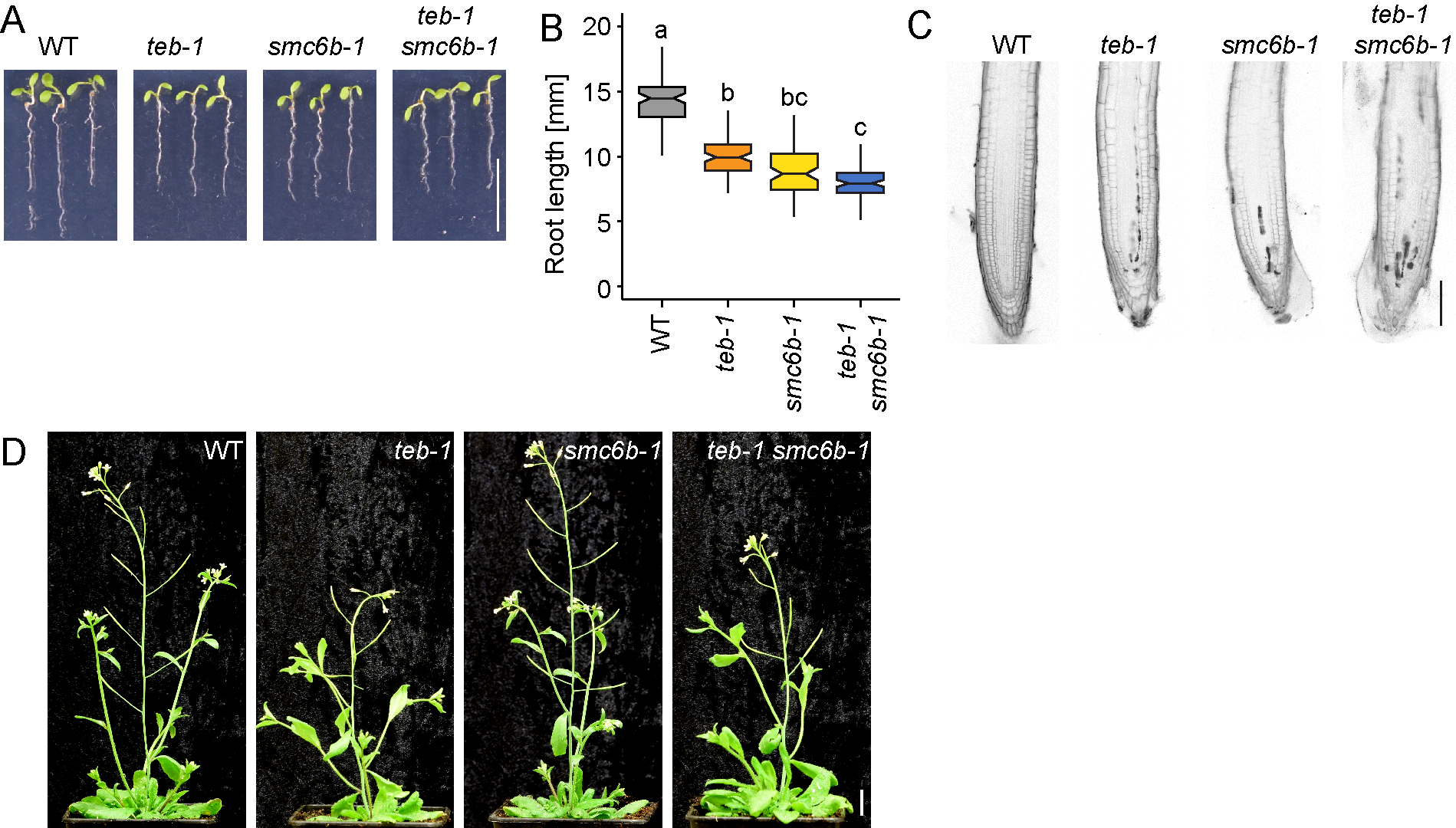


**Supplemental Figure 10 (related to Figure 6): Phenotypic analysis of *teb-1*, *smc6b-1,* and *teb-1 smc6b-1* mutants under normal conditions.** A, Root growth of 7-day-old WT, *teb-1*, *smc6b-1,* and *teb-1 smc6b-1*. Bar, 1 cm. B, Quantification of the root length of plants shown in (A). Data are means ± SD from three biological replicates, each with a minimum of 20 seedlings. Different lowercase letters indicate significant differences (*P*<0.05), according to one-way ANOVA followed by Tukey’s test. Source data are available in Supplemental Table10. C, Representative confocal microscopy images of plants shown in (A) stained with propidium iodide. Bar, 50 μm. D, Representative phenotypes of the six-week-old plants grown on soil. Scale bar, 1 cm.


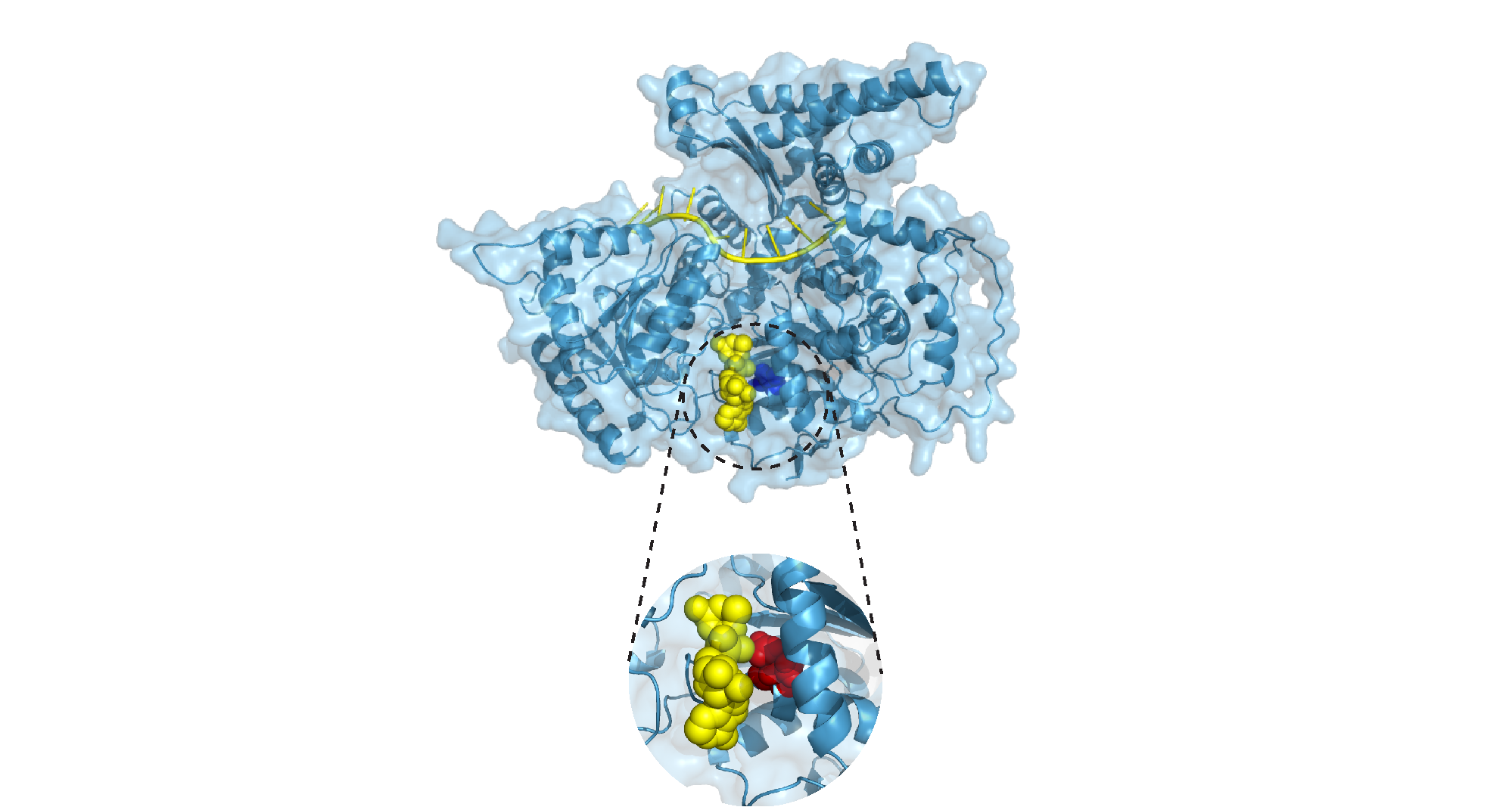
**Supplemental Figure 11 (related to Figure 2): Structural analysis of *hze2-4*.** Combined surface/cartoon representation (light/dark blue) of the Arabidopsis RTEL1 AlphaFold structure model (aa45-785). ATP (yellow spheres) and ssDNA (yellow ribbon) molecules were manually docked to the ATP-binding pocket and DNA-binding groove, respectively (based on 6FWS crystal structure; Cheng and Wigley, 2018). Small Thr90 residue (dark blue spheres; top panel) was substituted by the bulky Ile (red spheres in detail circular window below) obstructing ATP binding to the pocket.

**SUPPLEMENTAL TABLES**

**Supplemental Table 1: Source data for the statistical analyses to support Figure 1B.** Statistical significance was tested with the One-way ANOVA and is represented by an adjusted P-value compared to the respective WT control.

|  |  | **ZEB/mock (%)** | | | | | |
| --- | --- | --- | --- | --- | --- | --- | --- |
|  |  | **WT** | ***rtel1-1*** | ***hze2-1*** | ***hze2-2*** | ***hze2-3*** | ***hze2-4*** |
| Replicate 1 | | 46.3 | 25.8 | 20.0 | 25.3 | 25.3 | 17.3 |
| Replicate 2 | | 53.5 | 23.0 | 22.8 | 24.4 | 21.3 | 19.0 |
| Replicate 3 | | 39.4 | 21.2 | 18.6 | 26.0 | 23.6 | 16.0 |
| Average of replicates | | 46.4 | 23.3 | 20.5 | 25.2 | 23.4 | 17.4 |
| Standard deviation | | 7.0 | 2.3 | 2.1 | 0.8 | 2.0 | 1.5 |
| Statistical significance | WT |  | 0.000 | 0.000 | 0.000 | 0.000 | 0.000 |
|  | *rtel1-1* |  |  | 0.891 | 0.977 | 1.000 | 0.317 |
|  | *hze2-1* |  |  |  | 0.522 | 0.881 | 0.865 |
|  | *hze2-2* |  |  |  |  | 0.980 | 0.110 |
|  | *hze2-3* |  |  |  |  |  | 0.307 |
|  | *hze2-4* |  |  |  |  |  |  |

**Supplemental Table 2: Source data for the statistical analyses to support Supplemental Figure 7.** Statistical significance was tested with the One-way ANOVA and is represented by an adjusted P-value compared to the respective WT control.

|  |  | **CPT/mock (%)** | | | | | |
| --- | --- | --- | --- | --- | --- | --- | --- |
|  |  | **WT** | ***rtel1-1*** | ***hze2-1*** | ***hze2-2*** | ***hze2-3*** | ***hze2-4*** |
| Replicate 1 | | 67.8 | 66.6 | 60.1 | 52.5 | 46.0 | 60.3 |
| Replicate 2 | | 76.8 | 61.4 | 63.2 | 57.5 | 57.9 | 56.7 |
| Replicate 3 | | 55.6 | 52.9 | 50.3 | 52.8 | 45.8 | 56.0 |
| Average of replicates | | 66.7 | 60.3 | 57.9 | 54.2 | 49.9 | 57.7 |
| Standard deviation | | 10.6 | 6.9 | 6.7 | 2.8 | 6.9 | 2.3 |
| Statistical significance | WT |  | 0.838 | 0.599 | 0.270 | 0.079 | 0.578 |
|  | *rtel1-1* |  |  | 0.997 | 0.869 | 0.443 | 0.996 |
|  | *hze2-1* |  |  |  | 0.983 | 0.692 | 1.000 |
|  | *hze2-2* |  |  |  |  | 0.963 | 0.987 |
|  | *hze2-3* |  |  |  |  |  | 0.713 |
|  | *hze2-4* |  |  |  |  |  |  |
|  |  |  |  |  |  |  |  |
|  |  | **ICRF/mock (%)** | | | | | |
|  |  | **WT** | ***rtel1-1*** | ***hze2-1*** | ***hze2-2*** | ***hze2-3*** | ***hze2-4*** |
| Replicate 1 | | 74.0 | 64.9 | 66.8 | 68.3 | 71.7 | 73.3 |
| Replicate 2 | | 73.8 | 51.8 | 58.2 | 52.3 | 55.9 | 58.3 |
| Replicate 3 | | 62.0 | 50.9 | 60.0 | 49.3 | 56.6 | 61.8 |
| Average of replicates | | 69.9 | 55.9 | 61.6 | 56.6 | 61.4 | 64.5 |
| Standard deviation | | 6.8 | 7.9 | 4.5 | 10.2 | 8.9 | 7.9 |
| Statistical significance | WT |  | 0.314 | 0.787 | 0.363 | 0.767 | 0.953 |
|  | *rtel1-1* |  |  | 0.941 | 1.000 | 0.951 | 0.762 |
|  | *hze2-1* |  |  |  | 0.966 | 1.000 | 0.997 |
|  | *hze2-2* |  |  |  |  | 0.972 | 0.818 |
|  | *hze2-3* |  |  |  |  |  | 0.996 |
|  | *hze2-4* |  |  |  |  |  |  |

**Supplemental Table 3: Source data for the statistical analyses to support Figure 2.** Statistical significance was tested with the Kruskall-Wallis H-test with post hoc Conover-Iman test of multiple comparisons with the Benjamini-Hochberg procedure (P < ½ a, a = 0.05).

|  | **Synthesis-dependent strand annealing (SDSA)** | | | | | | |
| --- | --- | --- | --- | --- | --- | --- | --- |
|  |  | **mock** | | | **ZEB** | | |
| nr. of events | | **WT** | ***hze2-1*** | ***hze2-3*** | **WT** | ***hze2-1*** | ***hze2-3*** |
| Replicate 1 | | 0.1 | 0.9 | 0.7 | 0.6 | 1.0 | 0.6 |
| Replicate 2 | | 0.0 | 1.0 | 0.6 | 0.8 | 1.0 | 0.9 |
| Replicate 3 | | 0.0 | 0.8 | 0.7 | 1.0 | 1.0 | 0.5 |
| Average of replicates | | 0.0 | 0.9 | 0.7 | 0.8 | 1.0 | 0.7 |
| Standard deviation | | 0.0 | 0.1 | 0.0 | 0.2 | 0.0 | 0.2 |
| Statistical significance | WT mock |  | 0.000 | 0.000 | 0.000 | 0.000 | 0.000 |
|  | *hze2-1* mock |  |  | 0.067 | 0.324 | 0.213 | 0.122 |
|  | *hze2-3* mock |  |  |  | 0.142 | 0.009 | 0.363 |
|  | WT ZEB |  |  |  |  | 0.122 | 0.226 |
|  | *hze2-1* ZEB |  |  |  |  |  | 0.021 |
|  |  |  |  |  |  |  |  |
|  | **Single strand annealing (SSA)** | | | | | | |
|  |  | **mock** | | | **ZEB** | | |
| nr. of events | | **WT** | ***hze2-1*** | ***hze2-3*** | **WT** | ***hze2-1*** | ***hze2-3*** |
| Replicate 1 | | 3.0 | 3.3 | 3.7 | 6.9 | 4.0 | 6.2 |
| Replicate 2 | | 2.3 | 3.5 | 2.5 | 7.1 | 4.9 | 8.2 |
| Average of replicates | | 2.6 | 3.4 | 3.1 | 7.0 | 4.5 | 7.2 |
| Standard deviation | | 0.5 | 0.1 | 0.8 | 0.2 | 0.7 | 1.4 |
| Statistical significance | WT mock |  | 0.142 | 0.377 | 0.000 | 0.000 | 0.000 |
|  | *hze2-1* mock |  |  | 0.250 | 0.000 | 0.000 | 0.000 |
|  | *hze2-3* mock |  |  |  | 0.000 | 0.000 | 0.000 |
|  | WT ZEB |  |  |  |  | 0.000 | 0.449 |
|  | *hze2-1* ZEB |  |  |  |  |  | 0.000 |

**Supplemental Table 4: Source data for the statistical analyses to support Figure 3.** Statistical significance was tested with the One-way ANOVA and is represented by an adjusted P-value compared to the respective WT control.

|  |  | **ZEB/mock (%)** | | |
| --- | --- | --- | --- | --- |
|  |  | **WT** | ***teb-1*** | ***hze3-1*** |
| Replicate 1 | | 63.2 | 35.4 | 42.8 |
| Replicate 2 | | 58.9 | 40.2 | 28.5 |
| Replicate 3 | | 65.6 | 38.2 | 27.8 |
| Average of replicates | | 62.6 | 37.9 | 33.0 |
| Standard deviation | | 3.4 | 2.4 | 8.5 |
| Statistical significance | WT |  | 0.004 | 0.001 |
|  | *teb-1* |  |  | 0.549 |
|  |  |  |  |  |
|  |  | **CPT/mock (%)** | | |
|  |  | **WT** | ***teb-1*** | ***hze3-1*** |
| Replicate 1 | | 32.9 | 22.2 | 25.3 |
| Replicate 2 | | 46.0 | 29.2 | 23.7 |
| Replicate 3 | | 62.2 | 28.4 | 22.7 |
| Average of replicates | | 47.0 | 26.6 | 23.9 |
| Standard deviation | | 14.7 | 3.8 | 1.3 |
| Statistical significance | WT |  | 0.066 | 0.042 |
|  | *teb-1* |  |  | 0.926 |
|  |  |  |  |  |
|  |  | **ICRF/mock (%)** | | |
|  |  | **WT** | ***teb-1*** | ***hze3-1*** |
| Replicate 1 | | 85.7 | 50.3 | 52.2 |
| Replicate 2 | | 95.2 | 47.5 | 42.8 |
| Replicate 3 | | 80.6 | 38.6 | 41.8 |
| Average of replicates | | 87.2 | 45.5 | 45.6 |
| Standard deviation | | 7.4 | 6.1 | 5.7 |
| Statistical significance | WT |  | 0.001 | 0.001 |
|  | *teb-1* |  |  | 1.000 |

**Supplemental Table 5: Source data to support Figure 4.** Statistical significance was tested with a chi-square test.

|  | **Seeds in siliques** | | **Standard deviation** | **Chi-square test** | |
| --- | --- | --- | --- | --- | --- |
| **Genotype** | **aborted** | **normal** |  | **to WT** | **to *rtel1-1*** |
| WT | 0.2 | 99.8 | 0.5 |  | 0.055 |
| *teb-1* | 2.1 | 97.9 | 2.8 | 0.000 | 0.008 |
| *rtel1-1* | 7.0 | 93.0 | 3.5 | 0.000 |  |
| *rtel1-1 teb-1/+ nr. 13* | 36.1 | 63.9 | 10.7 | 0.000 | 0.000 |
| *rtel1-1 teb-1/+ nr. 7* | 37.6 | 62.4 | 8.3 | 0.000 | 0.000 |
| *rtel1-1 teb-1/+ nr. 23* | 33.8 | 66.2 | 5.3 | 0.000 | 0.000 |

**Supplemental Table 6: Source data to support Figure 5B.** Statistical significance was tested with the One-way ANOVA and is represented by an adjusted P-value.

| **Root length [mm]** | | | | | |
| --- | --- | --- | --- | --- | --- |
|  |  | **WT** | ***rtel1-1*** | ***smc6b-1*** | ***rtel1-1 smc6b-1*** |
| Replicate 1 | | 11.7 | 9.9 | 9.8 | 5.7 |
| Replicate 2 | | 11.7 | 10.0 | 9.9 | 6.7 |
| Replicate 3 | | 12.2 | 10.3 | 11.3 | 6.7 |
| Average of replicates | | 11.9 | 10.1 | 10.3 | 6.4 |
| Standard deviation | | 0.3 | 0.2 | 0.9 | 0.6 |
| Statistical significance | WT |  | 0.000 | 0.000 | 0.000 |
|  | *rtel1-1* |  |  | 0.657 | 0.000 |
|  | *smc6b-1* |  |  |  | 0.000 |

**Supplemental Table 7: Source data to support Figure 5D.** Statistical significance was tested with the One-way ANOVA and is represented by an adjusted P-value.

| **Meristem length [µm]** | | | | | |
| --- | --- | --- | --- | --- | --- |
|  |  | **WT** | ***rtel1-1*** | ***smc6b-1*** | ***rtel1-1 smc6b-1*** |
| Meristem length [µm] |  | 272.7 | 196.3 | 229.1 | 173.8 |
| Standard deviation | | 25.4 | 24.2 | 25.3 | 19.5 |
| Statistical significance | WT |  | 0.000 | 0.000 | 0.000 |
|  | *rtel1-1* |  |  | 0.001 | 0.013 |
|  | *smc6b-1* |  |  |  | 0.000 |

**Supplemental Table 8: Source data to support Supplemental Figure 9A**. Statistical significance was tested with the One-way ANOVA and is represented by an adjusted P-value.

| **Meristem cell number** | | | | | |
| --- | --- | --- | --- | --- | --- |
|  |  | **WT** | ***rtel1-1*** | ***smc6b-1*** | ***rtel1-1 smc6b-1*** |
| Number of cells |  | 26.0 | 17.3 | 21.2 | 14.9 |
| Standard deviation | | 1.9 | 2.0 | 2.3 | 1.6 |
| Statistical significance | WT |  | 0.000 | 0.000 | 0.000 |
|  | *rtel1-1* |  |  | 0.001 | 0.013 |
|  | *smc6b-1* |  |  |  | 0.000 |

**Supplemental Table 9: Source data to support Figure 5E.** Statistical significance was tested with the One-way ANOVA and is represented by an adjusted P-value compared to the respective WT control.

| **Number of dead cells adjacent to stele initials** | | | | | |
| --- | --- | --- | --- | --- | --- |
|  | | WT | *smc6b-1* | *rtel1-1* | *smc6b-1 rtel1-1* |
| Replicate 1 | | 0.091 | 0.900 | 1.091 | 2.071 |
| Replicate 2 | | 0.095 | 0.563 | 1.667 | 2.381 |
| Average of replicates | | 0.093 | 0.731 | 1.379 | 2.226 |
| Standard deviation | | 0.003 | 0.239 | 0.407 | 0.219 |
| Statistical significance | WT |  | 0.052 | 0.000 | 0.000 |
|  | *smc6b-1* |  |  | 0.006 | 0.000 |
|  | *rtel1-1* |  |  |  | 0.002 |
|  | *smc6b-1 rtel1-1* |  |  |  |  |

**Supplemental Table 10: Source data to support Supplemental Figure 10B.** Statistical significance was tested with the One-way ANOVA and is represented by an adjusted P-value.

| **Root length [mm]** | | | | | |
| --- | --- | --- | --- | --- | --- |
|  |  | **WT** | ***teb-1*** | ***smc6b-1*** | ***teb-1 smc6b-1*** |
| Replicate 1 | | 14.7 | 8.6 | 10.1 | 7.5 |
| Replicate 2 | | 14.1 | 7.4 | 9.7 | 7.1 |
| Replicate 3 | | 15.5 | 8.8 | 10.8 | 8.0 |
| Replicate 4 | | 13.1 | 10.7 | 9.2 | 8.5 |
| Average of replicates | | 14.3 | 8.9 | 9.9 | 7.8 |
| Standard deviation | | 1.0 | 1.4 | 0.7 | 0.6 |
| Statistical significance | WT |  | 0.000 | 0.000 | 0.000 |
|  | *teb-1* |  |  | 0.000 | 0.000 |
|  | *smc6b-1* |  |  |  | 0.001 |

**Supplemental Table 11: Source data to support Figure 6B.** Statistical significance was tested with the One-way ANOVA and is represented by an adjusted P-value.

| **ZEB/mock [%]** | | | | | |
| --- | --- | --- | --- | --- | --- |
|  |  | **WT** | ***teb-1*** | ***smc6b-1*** | ***teb-1 smc6b-1*** |
| Replicate 1 | | 77.8 | 57.0 | 42.0 | 44.9 |
| Replicate 2 | | 88.9 | 55.0 | 48.7 | 46.3 |
| Replicate 3 | | 80.2 | 52.0 | 42.0 | 41.5 |
| Average of replicates | | 82.3 | 54.7 | 44.2 | 44.3 |
| Standard deviation | | 5.8 | 2.5 | 3.8 | 2.5 |
| Statistical significance | WT |  | 0.000 | 0.000 | 0.000 |
|  | *teb-1* |  |  | 0.046 | 0.046 |
|  | *smc6b-1* |  |  |  | 1.000 |

**Supplemental Table 12: Source data to support Figure 6D.** Statistical significance was tested with the One-way ANOVA and is represented by an adjusted P-value.

| **ZEB/mock [%]** | | | | | |
| --- | --- | --- | --- | --- | --- |
|  |  | **WT** | ***rtel1-1*** | ***smc6b-1*** | ***rtel1-1 smc6b-1*** |
| Replicate 1 | | 77.8 | 66.8 | 42.0 | 55.2 |
| Replicate 2 | | 88.9 | 80.9 | 48.7 | 55.0 |
| Replicate 3 | | 80.2 | 67.0 | 42.0 | 48.8 |
| Average of replicates | | 82.3 | 71.6 | 44.2 | 53.0 |
| Standard deviation | | 5.8 | 8.1 | 3.8 | 3.7 |
| Statistical significance | WT |  | 0.173 | 0.000 | 0.001 |
|  | *rtel1-1* |  |  | 0.002 | 0.016 |
|  | *smc6b-1* |  |  |  | 0.304 |

**Supplemental Table 13. Primers used in this study.**

| **Target** | **Primer name** | **Sequence 5' to 3'** | **Application** |
| --- | --- | --- | --- |
| *RTEL1* | gRTEL2_F | GGGTTACCAAACGATTATAC | genotyping *rtel1-1* fwd; validation *hze2-6* gDNA |
| *RTEL1* | gRTEL2_R | CGACACAGAATATAAAGAACA | genotyping *rtel1-1* rev; validation *hze2-6* gDNA, cDNA |
| *SMC6B* | ET0035 | AGCTTCAACGTGAAATCATGG | genotyping *smc6b-1* fwd |
| *SMC6B* | ET0036 | CTAGACAACATGTCATACCGGG | genotyping *smc6b-1* rev |
| T-DNA SALK | LB_AP1 | ACTGGAACAACACTCAACCCTATCT | genotyping SALK T-DNA lines |
| *TEB* | ET0328 | TTTCTAGGTCGAGTGTCTTCAG | genotyping *teb-1 -* fwd (Ishida *et al*., 2006) |
| *TEB* | ET0329 | TTACATCCTGTGGTCTGTTTAGAG | genotyping *teb-1 -* deletion (Ishida *et al*., 2006) |
| *TEB* | ET0330 | ATGAGCAGGATCAGCTTTGCC | genotyping *teb-1 -* rev (Ishida *et al*., 2006) |
| *RTEL1* | ET0319 | GGCGTACCAGTCTCAGATCA | validation *hze2-3*, *hze2-4* gDNA, cDNA |
| *RTEL1* | ET0320 | CGGGACGCATAAACTATGGTG | validation *hze2-3*, *hze2-4* gDNA, cDNA |
| *RTEL1* | ET0131 | AAGGACTGCTGATATTTTTCC | validation *hze2-2*, *hze2-5* gDNA |
| *RTEL1* | KP81 | ACAACTTGCAGATGTTAAGA | validation *hze2-2*, *hze2-5* gDNA |
| *RTEL1* | ET0152 | CTTGACGTTAGGTCGTATCCA | validation *hze2-1* gDNA, cDNA; *hze2-2*, *hze2-5* cDNA |
| *RTEL1* | ET0151 | GTCAGCGAAGGACTGGATTT | validation *hze2-1* gDNA, cDNA |
| *RTEL1* | ET0129 | TGAAAGTCAACTGGACTAAT | validation *hze2-6* cDNA |
| *TEB* | ET0332 | ATCGTGGTGTAAGATCTGTTTC | validation *hze3-1* gDNA, cDNA |
| *TEB* | ET0334 | TTCTCGGCACAAATGGATAC | validation *hze3-1* gDNA, cDNA |
